# Tire Deformation-Based Regulation of Braking Torque in Manual Wheelchairs Equipped with Reverse Locking Modules

**DOI:** 10.1371/journal.pone.0325504

**Published:** 2025-06-17

**Authors:** Bartosz Wieczorek, Łukasz Warguła, Marcin Giedrowicz

**Affiliations:** 1 Institute of Machine Design, Faculty of Mechanical Engineering, Poznan University of Technology, Poznan, Poland; 2 Institute of Architecture and Spatial Planning, Faculty of Architecture, Poznan University of Technology, Poznan, Poland; Tecnológico de Monterrey, MEXICO

## Abstract

Moving in a manual wheelchair involves overcoming various architectural and terrain barriers. One of the obstacles that most burdens the muscular system and generates a high risk of instability is the climb up a slope. This article presents a comprehensive regulation method that allows for achieving the desired braking torque of the locking module based solely on tire deformation measurements, rather than the previously used contact force. To address the research problem, a research method was developed, consisting of three experimental tests and one mathematical analysis. The experiments included the measurement of the sliding force moment (E1), braking torque (E2), and tire deformation (E3). Using these methods, a measurement procedure was formulated to allow the measurement of the braking torque generated by the reverse locking module through tire deformation. Research on braking torque M_h_ showed that for wheelchairs with 24’’x1’’ wheels and a tire pressure of 4-7 bar, tire deformation e_T_, depending on the diameter of the pressing roller, ranges from mm to mm. For a constant roller diameter of 70 mm, to achieve a torque of 7.5 Nm, the deformation was mm, and for 12 Nm – mm. The sliding force FZ increased by 57% with the user’s mass rising from 50 kg to 90 kg (from N to N at a pressure of 7 bar). ANOVA analysis confirmed that both the nominal contact force F_dN_ and the diameter of the roller d_r_ had a significant impact on the braking torque M_h_. Verification of the developed mathematical model of braking torque as a function of tire deformation showed an error range of 3% to 7%.

## 1. Introduction

Manual wheelchairs, especially those with continuous drive systems, remain consistently popular among individuals with mobility impairments. Their versatility and mobility contribute to improving the users’ quality of life, enabling them to maintain physical activity. Regular use of such a drive system has a positive impact on rehabilitation, as it helps maintain physical fitness and independence [1–3]. However, manual propulsion comes with certain limitations, which are particularly evident in challenging terrain conditions [4,5]. The user’s physical predispositions, as well as the terrain, can significantly hinder movement, especially when it comes to inclines. Climbing a slope in a wheelchair presents a complex problem from a physics perspective, particularly when analyzing the forces acting on the wheelchair and their impact on the user’s stability. Key physical phenomena that need to be considered include gravity, friction, and the moments of driving forces [6,7]. When analyzing the risks associated with ascending a slope in a wheelchair, it is essential to consider scenarios where manual propulsion may be halted, such as due to extreme user fatigue. This situation may lead to the wheelchair rolling backward, posing a significant threat to the user’s health and safety. Additionally, user fatigue can weaken their ability to control the wheelchair [8–10].

In the context of the discussed risks associated with climbing slopes with a manual wheelchair, the use of additional modules to assist the manual drive becomes crucial for ensuring user safety and comfort. Examples of such modules include hybrid manual-electric drives. [11–14] and the reverse locking module [15–17]. Both solutions offer significant benefits that can greatly improve the mobility and stability of wheelchairs. The reverse locking module is a small component that can be implemented in any manually powered wheelchair. It operates on the principle of a braking mechanism, which activates when the gravitational force begins to dominate over the frictional force.

The use of the reverse locking module in the drive system of a manually powered wheelchair requires its integration with the wheelchair’s drive wheel by pressing a roller against the drive wheel. This action involves the deformation of the wheelchair’s tire; however, the applied force is necessary to generate friction strong enough to block the wheelchair from rolling down a slope. Unfortunately, excessive tire deformation leads to an increase in the energy required to roll the drive wheel over the roller. Furthermore, tire deformation also contributes to higher noise levels [18] and increased tire wear [19]. In this context, there is a need for precise regulation of the anti-rollback module’s pressure on the wheelchair wheel, ensuring that the adjusted pressure guarantees the module’s functionality without increasing the negative properties of the friction connection that generates tire deformation. Based on the above, it is necessary to regulate the force applied by the reverse locking roller to the wheelchair’s wheel. This regulation must be performed individually and depends on several factors related to the user’s individual traits and the geometric characteristics of the wheelchair in use. Due to the design of the entire system, it is not possible to use a traditional force sensor. Therefore, it is necessary to determine a method for estimating the braking torque generated by the reverse locking module [20] based on measurements that can be performed on the wheelchair without altering its structure.

The above guidelines have created a research problem consisting of developing a converter that allows for regulating the braking torque generated by the pressure of the roller on the drive wheel based on the measurement of the tire deformation on the wheelchair’s wheel. Current studies do not address this topic and focus on the impact of braking on tire deformation as a process of deformation resulting from the action of inertia forces. [21–23]. In the described device, the braking moment M_h_ is the result of the friction coupling between the anti-rollback module roller and the drive wheel [24], similar to what occurs in dynamometers used for vehicle testing [25–27]. In the discussed device, M_h_ refers to the moment at which the wheelchair wheel moves out of static equilibrium[28] and begins to rotate. Referring to the deformation of the wheelchair tire [29] the measured value is the depth of the anti-rollback module roller’s indentation into the wheelchair tire e_T_. This issue is an area that requires experimental research, as available studies address topics such as ground deformation [30] or deformations related to pressing a flat surface against the tire [31,32].

Based on the above research problem, a hypothesis was formulated stating that it is possible to develop a functional mathematical model which, given a known pressure in the wheelchair tire, allows assigning a specific value of tire deformation to the corresponding value of generated braking torque. The main objective of this study is to develop a generalized analytical model that links the deformation of the wheelchair’s drive wheel tire caused by the pressing force of the anti-rollback module’s roller with the value of the resulting braking torque.

To achieve this analytical objective, a series of experiments were conducted, each constituting a separate research task:

- determination of the sliding moment value of a wheelchair as a function of the incline angle
- determination of the relationship between the pressing force of the anti-rollback roller and tire deformation
- determination of the relationship between the braking torque and the pressing force of the anti-rollback roller acting on the wheelchair wheel

The results obtained from these three experiments form the basis for deriving the analytical model, which enables the estimation of braking torque based solely on tire deformation measurements without the need to interfere with the wheelchair’s construction.

## 2. Method and materials

**Fig 1:**
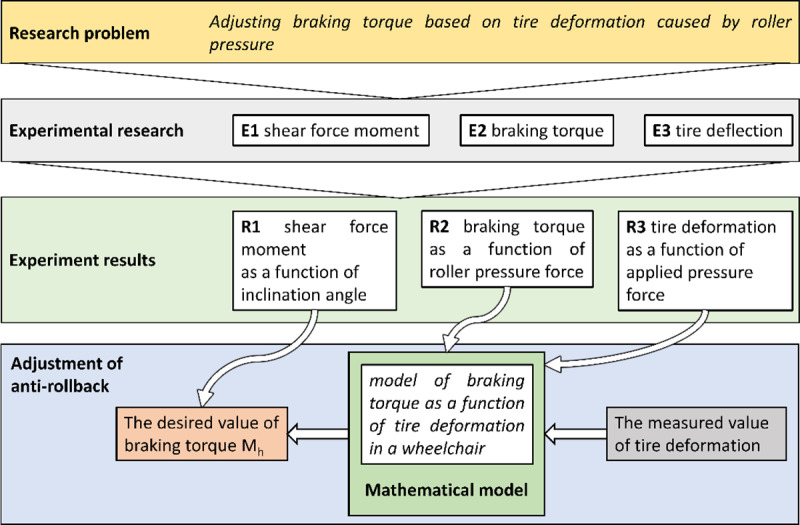
The algorithm for achieving the research objectives illustrating the process of developing a mathematical model of the braking moment as a function of tire deformation.

The solution to the research problem required the development of a research method consisting of three independent experimental studies conducted at different research stations, as well as one analytical method for processing data obtained through experimentation (Fig 1). Among the experimental studies conducted were: measurement of the sliding force moment (E1), measurement of the braking moment (E2), and measurement of tire deformation (E3). The measurement of the sliding force moment allowed for determining the effect of the incline angle on the sliding force that generates the moment causing the wheelchair to slide. The result of this study was the determination of the sliding force moment F_Z_ as a function of the incline angle (R1). Defining this relationship enabled the analytical determination of the minimum braking moment M_h_ required to prevent the wheelchair from sliding due to gravity. The braking moment study allowed for determining its value as a function of the pressure force F_d_ exerted by the reverse lock roller on the drive wheel (R2). In the context of the adopted research problem, it was necessary to conduct further studies to define the tire deformation function as a function of the pressure force exerted by the reverse lock roller on the drive wheel (R3). Taking into account the results of the two previous studies (R2 and R3), it was possible to derive a mathematical model of the braking moment as a function of the wheelchair tire deformation. This model allows for substituting the measured tire deformation and calculating the braking moment M_h_.

### 2.1 Materials and research stations

**Fig 2:**
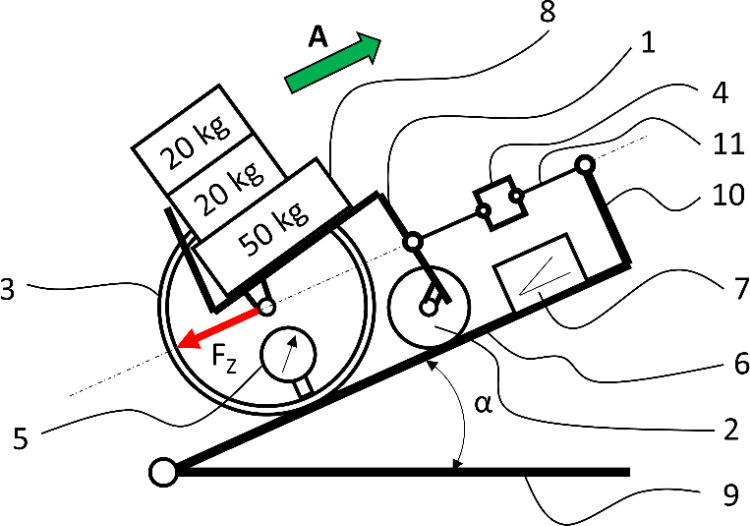
Schematic of the setup used to measure the sliding force of a wheelchair on an incline.

The measurement of the sliding force F_Z_ under real-world conditions was conducted at a research station (Fig 2) consisting of a Vermeiren V300 wheelchair weighing 17.7 kg [1], equipped with solid front wheels [2] and pneumatic rear wheels with a diameter of 24” [3]. The wheelchair was positioned on a polished oak inclined plane [6], with an adjustable incline angle α relative to the ground [9]. The setup included a pressure measurement system [5] for the wheelchair tires, an inclinometer [7] to measure the wheelchair’s incline angle α, and a force sensor [4] to measure the sliding force F_Z_. The force sensor was connected to a stationary base [10] and the wheelchair frame via a steel cable [11] arranged in a measurement position parallel to the surface of the inclined plane [6]. The tested wheelchair was sequentially loaded with masses [8], arranged to approximate the distribution of a person’s mass while seated in the wheelchair. The described setup met the basic guidelines of known and commonly used test stations for measuring friction force using an inclined plane [33].

The study used a force sensor in the form of a Zemic H3 strain gauge with a measurement range of 200 kg and an absolute measurement error of 0.02%. The incline angle was measured using a KIONIX KX023 inertial sensor with a resolution of 0.009576801 m/s² and a measurement range of 78.4532 m/s². The pressure in the drive wheels of the tested wheelchair was measured using an analog manometer with a range of 15 bar, which had a measurement error of 2% for the analyzed pressure range (6–10 bar)

**Fig 3:**
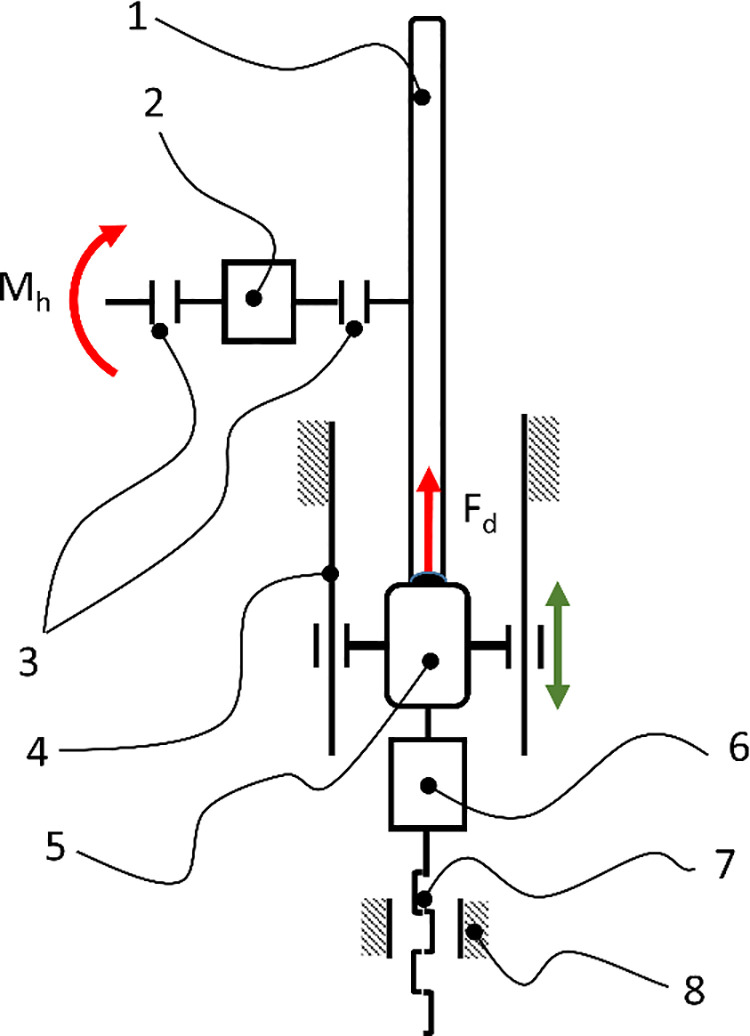
Schematic of the research setup used to measure the braking moment for the locked and pressed reverse lock roller module.

The braking moment was measured using a proprietary research setup designed for laboratory measurement of motion resistance. The setup was developed according to the guidelines used in the measurement of torque and power [34] in devices such as engine dynamometers [35]. Using the developed measurement system (Fig 3), simultaneous measurement of the pressure force exerted by the reverse lock roller on the wheelchair wheel F_d_ and the braking moment M_h_ was carried out. The main component of the setup is a torque meter [2] from HBM, model 1-T20WN/100NM, with an accuracy class of 0.2. The torque meter is equipped with two shafts, to which, using couplings [3], a moment M_h_ was applied, forcing the rotation of the wheelchair wheel [1] mounted on the opposite side of the torque meter. The reverse lock roller [5], which had a blocked rotation capability, was pressed against the wheelchair wheel. The pressing was done using linear guides [4] and a screw [7] rotating relative to a stationary nut [8]. The pressure force was measured using a force sensor [6] from Zemic, model Tensometer H3-C3-100 kg, with a measurement range of 1000 N and an absolute error of 0.02%. The force sensor [6] served as a connector between the movable reverse lock roller [5] and the pressing force exerted by the screw [7].

**Fig 4:**
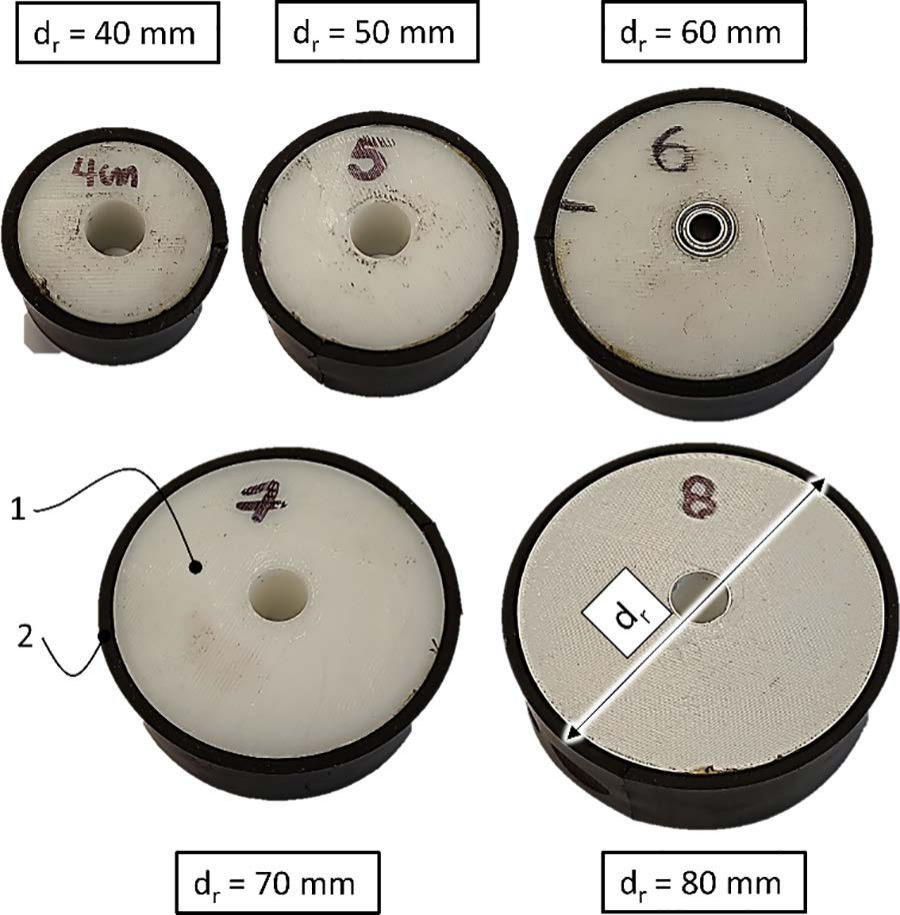
The studied reverse lock module rollers, with the variable diameter and component elements indicated, where dr – the nominal diameter of the roller, 1 – core, 2 – rubber layer.

The tests were conducted for a wheelchair wheel equipped with a Schwalbe Rightrun inner tube tire, with a diameter of 24“ and a width of 1”. The pressure p in the tire was maintained constant at 6 bar, which was the lower nominal value for the high-pressure tire used. The variable technical element during the testing procedure was the diameter of the pressed roller d_r_, which varied as follows: 40 mm, 50 mm, 60 mm, 70 mm, and 80 mm (Fig 4). The roller consisted of a solid PLA core [1], and its outer surface in contact with the wheel was covered with a 3 mm layer of butadiene-styrene rubber [2].

**Fig 5:**
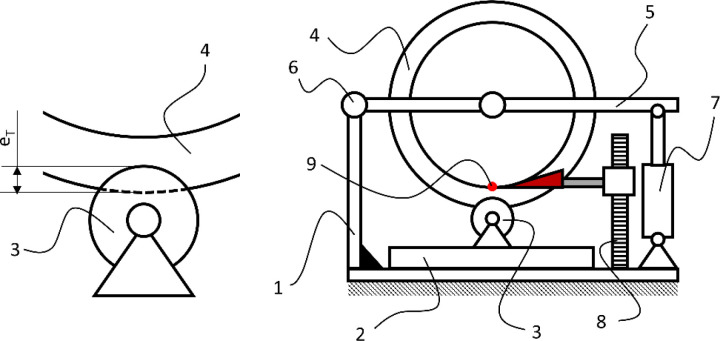
Schematic of the research setup used to study tire deformation as a function of the pressure force exerted by the reverse lock roller module.

The measurement of tire deformation as a function of the pressure force Fd exerted by the roller on its surface was carried out at a research station (Fig 5), which is the subject of a patent application with the Polish Patent Office (P.447196). The setup consisted of a frame [1] on which a scale [2] with a fixed and stationary roller [3] was placed. The scale used had a measurement range of 200 kg and a measurement accuracy of 100 g. The wheelchair wheel [4] was mounted on a pivot arm [5]. This arm was supported at one end by a hinge joint [6] and at the other end by a linear actuator [7]. As the linear actuator shortened, the axis of rotation of the wheelchair wheel moved closer to the axis of rotation of the tested roller, resulting in tire deformation e_T_. The value of this deformation was measured using a dial gauge [8] with a measurement accuracy of 0.01 mm. The dial gauge was applied at the lowest point on the inner edge of the wheelchair wheel rim [9]. The reading from the dial gauge was zeroed for the contact point between the wheelchair wheel and the reverse lock roller, where the applied pressure force oscillated around 0 ± 10 N.

In the tire deformation study, five variants of the reverse lock roller module with different diameters dr were used, which were as follows: 30 mm, 40 mm, 50 mm, 60 mm, 70 mm, 80 mm, and 90 mm (Fig 4). The roller consisted of a solid PLA core [1], and its outer surface in contact with the wheel was covered with a 3 mm layer of butadiene-styrene rubber [2]. The wheel to which the roller was pressed consisted of a 24“ rim, onto which three types of tires were mounted: MBL Gazelle 24x1”, MBL SpeedLite 24x1”, and MBL TrailBlazer 24x1” (Table 1). These tires were chosen because their size is the most commonly used in wheelchairs for adults. Furthermore, the selected tire models represent different tread finishes and insert reinforcement sizes, adapted for use in various terrain conditions.

**Table 1:** Technical specifications of the tires used in the study.

	MBL Gazelle 24x1“	MBL SpeedLite 24x1“	MBL TrailBlazer 24x1“
Ilość warstw	2	2	2
Szerokość wkładki usztywniającej	15 mm	15 mm	18 mm
Wysokość wkładki usztywniającej	---	---	4,5 mm
Materiał warstw	tkany nylon SPL	guma naturalna PTR	guma naturalna PTR
Oplot	127 TPI	67 TPI	67 TPI
Zalecane ciśnienie	6–10 bar	6–10 bar	6–10 bar
Maksymalne obciążenie	70 kg	70 kg	70 kg

### 2.2 Measurement procedures

The complexity of the research objective required the development of three independent research procedures, each characterized by different dependent and independent variables. The method for measuring the sliding force F_Z_ involved applying different loads Q and incline angles α for a constant tire pressure p. The dependent variable in this method was the value of the sliding force F_Z_. The algorithms for the research procedure, consistent with the used measurement setup (Fig 2), consisted of the following steps:

- Step 1: Load the wheelchair with the required weight Q [8]
- Step 2: Check the tire pressure p or adjust it to the desired value using the manometer [5]
- Step 3: Adjust the wheelchair incline to the desired angle α
- Step 4: Move the wheelchair upward in the direction A, so that the steel cable [11] loosens
- Step 5: Lock the drive wheels [3]
- Step 6: Zero the force sensor [4]
- Step 7: Unlock the drive wheels [3] and slowly allow the wheelchair to slide until the steel cable [11] is fully tensioned
- Step 8: Read and archive the sliding force F_Z_ for the specified wheelchair load Q and incline angle α.

During the measurement, for each value of wheelchair load Q and incline angle α, six repetitions were performed, starting each from step 4 of the research procedure.

**Fig 6:**
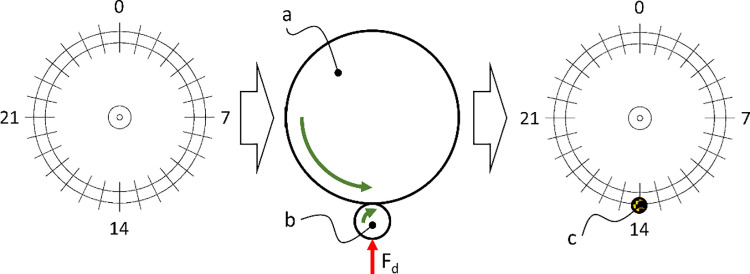
The procedure diagram for analyzing the curvature of the circle in order to determine the nominal point to which the initial value of the roller pressure force for the reverse lock module was applied. Description of symbols in the text.

The research procedure for measuring the braking torque M_h_ aimed to measure the maximum value of the torque applied to the wheelchair’s drive wheel, which was blocked by the pressed roller while remaining at rest. The value of this torque M_h_ was the dependent variable, while the independent variable was the pressing force Fd exerted by the roller on the wheelchair’s wheel. The methodology first involved performing an analysis of the wheel’s curvature to determine the nominal point, which served as the starting position where the constant pressing force was set (Fig 6). According to the procedure, the first step was to mark 28 measurement points on the tested wheelchair wheel, corresponding to the points where the spokes met the rim. Next, the roller (b) was pressed against the wheelchair’s wheel (a) with a constant value of pressing force F_d_. The wheelchair wheel was then set in motion, resulting in a change in the value of the pressing force F_d_ caused by deviations in the roundness of the tested wheel. After completing a full rotation of the wheel, the values of the pressing force F_d_ were analyzed at the marked measurement points, and the minimum value of the pressing force F_d_ was identified. At the location where the minimum value of F_d_ occurred, the nominal point (c) was marked. This nominal point was then used as the starting position in subsequent measurements, and the initial pressing force F_d_ was applied to the tested roller.

During the measurement test M_h_, each of the tested rollers of the reverse lock module was pressed against the wheelchair wheel at the nominal point on the wheel with a force F_d_ of: 5 N, 10 N, 15 N, 20 N, 25 N, 30 N, 35 N, 40 N. Then, with the reverse lock module roller immobilized, a torque was applied to the wheelchair wheel, increasing its value until the wheel was displaced from its static equilibrium. The torque increase was carried out under quasi-static conditions, minimizing the effects of inertial forces. This process was repeated for each designated measurement point on the wheel. As a result, the average braking torque M_h_ (2) was obtained as a function of the average pressing force F_d_ (3), where the averaging was done over one full rotation of the wheel, i.e., 28 measurement points.


$$M_{h}=\frac{\sum {\mathrm{\backslash nolimits}}_{i=1}^{n}M_{h}^{i}}{n}$$ (1)

$$F_{d}=\frac{\sum {\mathrm{\backslash nolimits}}_{i=1}^{n}F_{d}^{i}}{n}$$ (2)

Where: M_h_ – braking torque, the average over one full rotation of the wheelchair wheel, F_d_ – pressing force (the average over one full rotation of the wheel), i – any measurement point on the wheelchair wheel, n – the number of designated measurement points on the wheelchair wheel.

In the subsequent steps, for the measurement points determined in this way, the braking torque M_h_ was calculated as a function of the pressing force Fd of the reverse lock module roller. Such characteristics were determined for all five diameters of the reverse lock module rollers.

**Fig 7:**
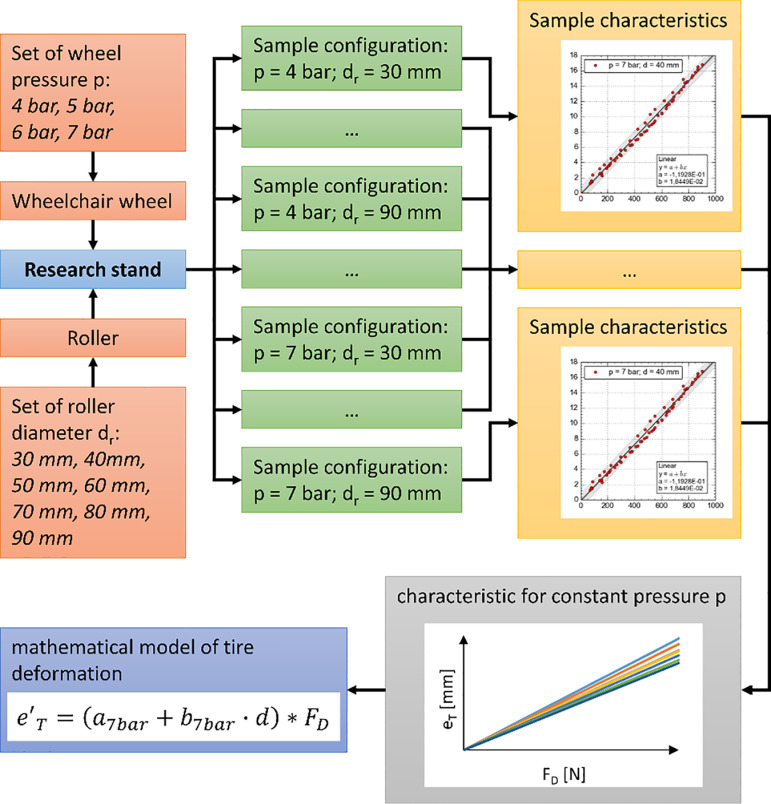
The algorithm of the research procedure along with the data processing workflow, where: d_r_ – diameter of the reverse locking module roller, F_d_ – pressing force of the roller, p – internal pressure of the wheelchair tire, a_7 bar_ – intercept of the deformation model’s slope coefficient, b_7 bar_ – slope coefficient dependent on the tire pressure value.

**Fig 8:**
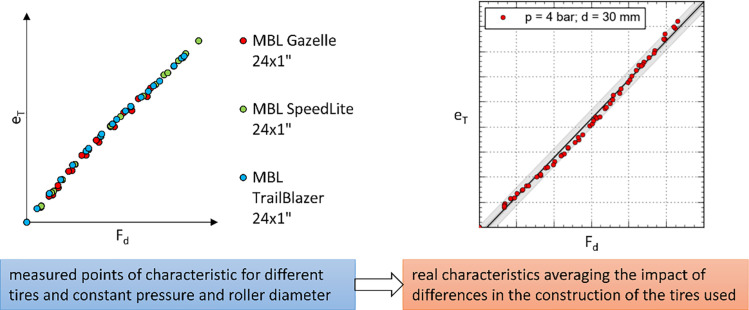
An example of linking the measurement points with the determined actual tire deformation characteristic (including three types of tires) for a constant tire pressure p and roller diameter d_r_.

The final study conducted focused on examining the deformation of a wheelchair tire caused by the pressure exerted by the roller of the reverse locking module. The research methodology (Fig 7) assumed that for each variant of tire inflation pressure p (ranging from 4 to 7 bar), all diameters dr of the reverse locking module roller were tested. For each configuration of pressure p and roller diameter d_r_, an experiment was carried out to determine the actual deformation characteristics of the tire e_T_ as a function of the pressing force F_d_ exerted by the reverse locking module roller on the wheelchair wheel. Each individual characteristic resulted from an experiment conducted with constant pressure p, a specific roller diameter d_r_, and each of the three tire types (Table 1) (Fig 8). For each tire, the minimum number of collected measurement points was no less than 20. Based on all the conducted experiments, the individual deformation curves were grouped according to the pressure value p used in the study. In this way, four mathematical models were developed to describe the tire deformation function depending on the diameter of the roller d_r_ and the pressing force F_d_ applied to the wheelchair wheel.

## 3. Results

### 3.1 Measurement of the sliding force as a function of the slope inclination angle

**Fig 9:**
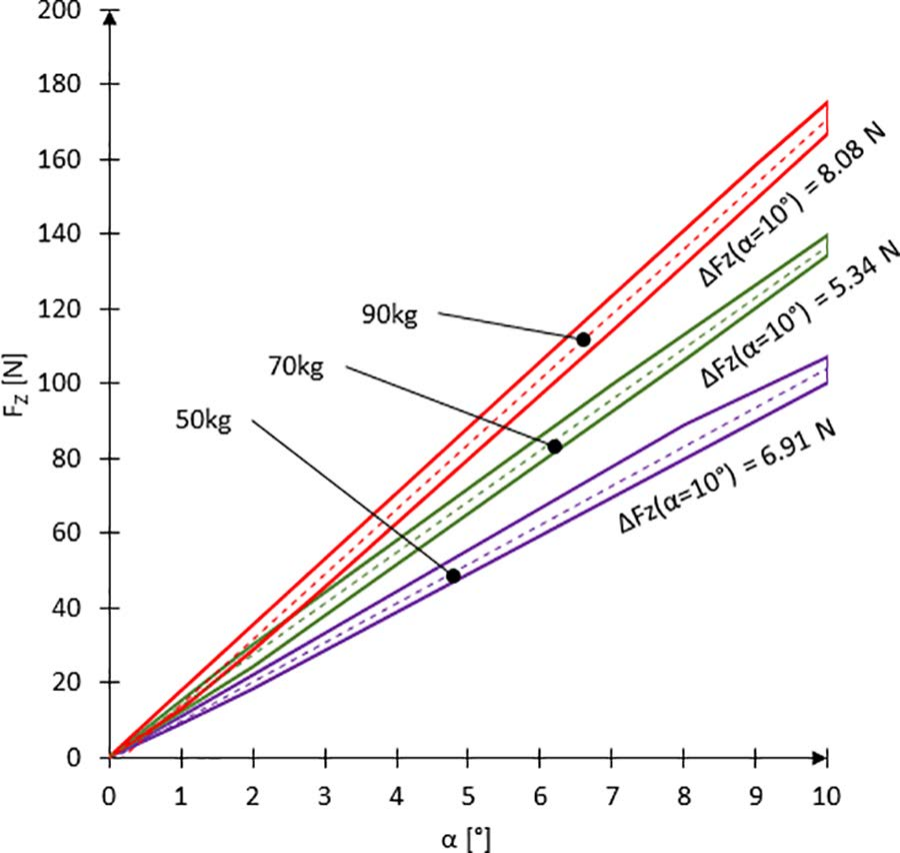
Graphs of the sliding force F_Z_ for user masses m = 50, 70, and 90 kg and tire pressures p ranging from 3 to 7 bar.

This section addresses the first experimental task, which aimed to determine the value of the sliding moment of a wheelchair on an inclined surface as a function of the ramp angle α. This allows estimation of the minimum braking torque necessary to prevent unintentional backward motion under gravity. The results of the sliding force F_Z_ tests (Fig 9) (S1 Appendix A) showed that the user’s mass m had the greatest influence on the force value. The minimum F_Z_ value at a slope inclination of α = 10° was recorded for a user mass of m = 50 kg and amounted to 100.67 N at a tire pressure of p = 7 bar. In contrast, the maximum F_Z_ value for the same slope angle was measured for a load of m = 90 kg, with a force of 175.33 N at a tire pressure of p = 3 bar. The difference in F_Z_ values resulting from changes in pressure is presented as the parameter ΔF_Z_. The ΔF_Z_ value reached its highest values at the extreme range of the tested slope angles and for the analyzed variants amounted to 6.91 N for m = 50 kg, 5.34 N for m = 70 kg, and 8.08 N for m = 90 kg.

**Fig 10:**
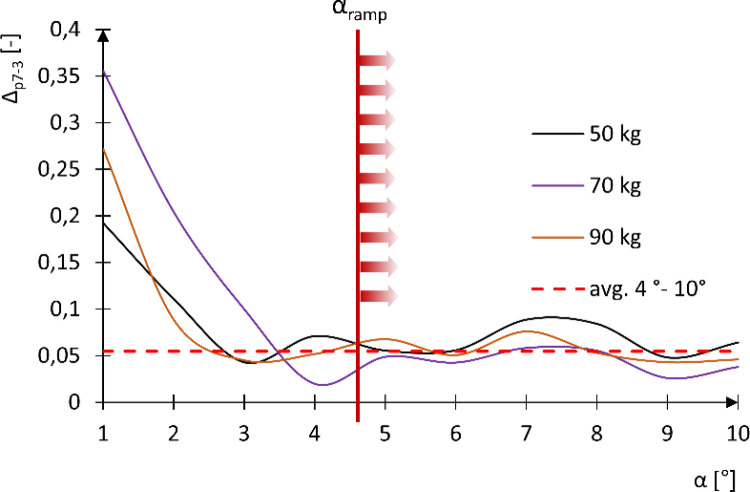
Graph of the percentage difference in sliding force F_Z_ between tire pressures of 3 and 7 bar (Δ_p7−3_) as a function of the slope inclination angle α.

Analyzing the above graphs and performing an ANOVA analysis (Table 2), it was concluded that the dominant factor affecting the value of the sliding force F_Z_ is the user’s mass m. The tire pressure value plays a secondary role and does not significantly influence changes in F_Z_. This is confirmed by the analysis of the percentage difference in F_Z_ between 7 and 3 bar tire pressure, denoted as Δ_p7−3_ (Fig 10). The analysis showed that regardless of user mass, the difference in F_Z_ between 7 and 3 bar pressure (Δ_p7−3_) is minimal. The highest values were observed for slope angles α ≤ 3.5°, reaching from 19% to 36%. This large percentage difference in F_Z_ is only noticeable at small inclination angles, where the gravitational force contribution is minor and the rolling resistance due to tire deformation plays a significant role. However, for the ramp inclination angle αramp of approximately 4.6°, which corresponds to typical access ramps in accordance with building standards, the Δ_p7−3_ value stabilizes at around 5.4%. It should be noted that for inclination angles α ≤ 3°, the value of the sliding force F_Z_ remains low, ranging from 29.58 N to 48.00 N. Therefore, a high percentage difference Δ_p7−3_ does not translate into a significant absolute difference in F_Z_ between 3 and 7 bar tire pressure. Nonetheless, in the context of using assistive devices for manual wheelchair propulsion—especially when overcoming terrain obstacles—the values of F_Z_ corresponding to the αramp inclination angle are particularly relevant.

**Table 2:** Results of the ANOVA analysis evaluating the statistical significance of variables on the value of the sliding force F_Z._

Source of Variation	df	F-statistic	p-value	Percentage Influence on F_Z_ (%)
m	2	45.67	0.0001	40.8
α	9	12.34	0.0012	18.8
p	6	5.67	0.0234	7.8

Considering the negligible influence of tire pressure on the value of the sliding force F_Z_ within the analyzed range of slope inclination angles (α_ramp_), this parameter was omitted as a variable in subsequent analyses. Instead, a function of the sliding force F_Z_ dependent solely on the slope inclination angle α was determined [3–5] (S2 Appendix B, Fig 5–7). A linear mathematical model was selected to describe this relationship, and the values of its parameters were calculated with a confidence level of p = 0.05.


$$\mathrm{\backslash [}F_{Z}^{m=50kg}\left(\alpha \right)=\left(10,416810\pm 0,080386\right)\alpha \mathrm{\backslash ]}$$ (3)

$$\mathrm{\backslash [}F_{Z}^{m=70kg}\left(\alpha \right)=\left(13,708646\pm 0,076627\right)\alpha \mathrm{\backslash ]}$$ (4)

$$\mathrm{\backslash [}F_{Z}^{m=90kg}\left(\alpha \right)=\left(16,943584\pm 0,127297\right)\alpha \mathrm{\backslash ]}$$ (5)

Given the diameter of the wheelchair’s drive wheel of 24” and the developed mathematical models, it was possible to determine the braking moment M_h_ at which the wheelchair begins to roll down a slope with a specified inclination angle. According to building regulations concerning slope gradients, it is assumed that ramps in public spaces have an inclination of 4.6°. Based on this, it was determined that the value of the braking moment M_h_ generated through friction between the reverse locking module roller and the wheelchair wheel should be: 7.5 Nm for a user mass of 50 kg, 10 Nm for a user mass of 70 kg, and 12 Nm for a user mass of 90 kg.

### 3.2 Measurement of the braking moment as a function of the pressing force of the roller on the drive wheel

**Fig 11:**
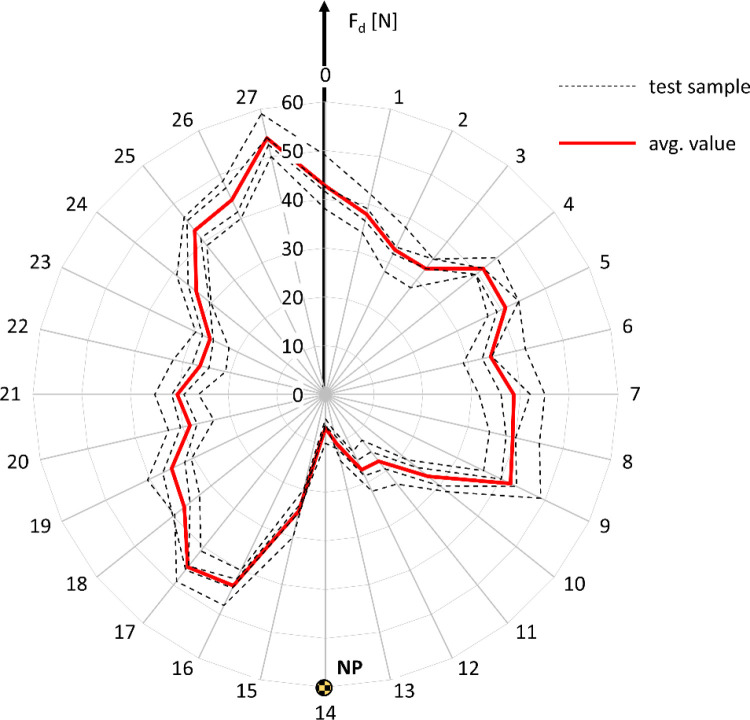
An example graph showing the variation in the pressing force F_d_ of the roller against the wheel, resulting from the out-of-roundness of the tested wheel, with the nominal point NP marked at the location of the minimum F_d_ value.

This section corresponds to the second experimental task, which involved determining the braking moment Mh as a function of the pressing force Fd exerted by the anti-rollback roller on the wheelchair drive wheel. These results provide the foundation for later linking braking torque to tire deformation. The procedure for determining the braking moment M_h_ as a function of the pressing force F_d_ of the roller on the drive wheel initially involved identifying the nominal point NP on the circumference of the wheelchair wheel (Fig 11).

The wheel curvature test revealed that, for a constant initial pressing force, the minimum F_d_ value was recorded at point 14 and amounted to 6.9 N ± 3.5 N. The maximum pressing force was measured at point 27 and reached 53.9 N ± 6.0 N. Based on the graph showing the variation in F_d_ caused by the wheel’s out-of-roundness, point 14 was identified as the nominal point NP. In subsequent tests, the nominal pressing force F_dN_ of the roller against the wheel will be applied at point 14. This force will be adjusted incrementally in 5 N steps, within the range from 5 N to 40 N.

**Fig 12:**
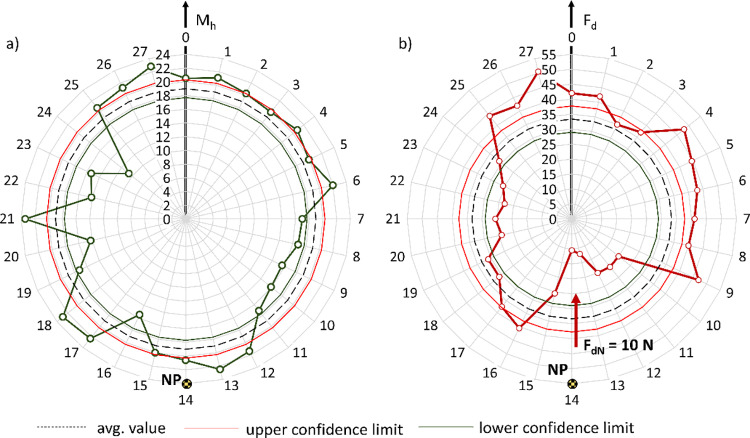
Characteristics of the variation in braking moment M_h_, expressed in Nm **(a)**, and pressing force F_d_, expressed in N **(b)**, resulting from the out-of-roundness of the wheelchair wheel, for the test of the reverse locking module roller with a diameter of d_r_ = 40 mm and a nominal pressing force F_dN_ = 10 N applied at point NP = 14.

Given the presence of significant deviations in the roundness of the wheelchair wheel, which influence the variation of the pressing force F_d_ relative to the set nominal value F_dN_, it was necessary to perform a study of the changes in the braking moment M_h_ over one full rotation of the drive wheel, while maintaining a constant nominal pressing force F_dN_ at the nominal point NP. Example results for the reverse locking module roller with a diameter of d_r_ = 40 mm and a nominal pressing force F_d_ applied at point NP = 14 are presented in Fig 12. Based on the data collected in this way, the average values of the braking moment M_h_ and pressing force F_d_ were determined over one full wheel rotation at a constant nominal force setting F_dN_.

**Fig 13:**
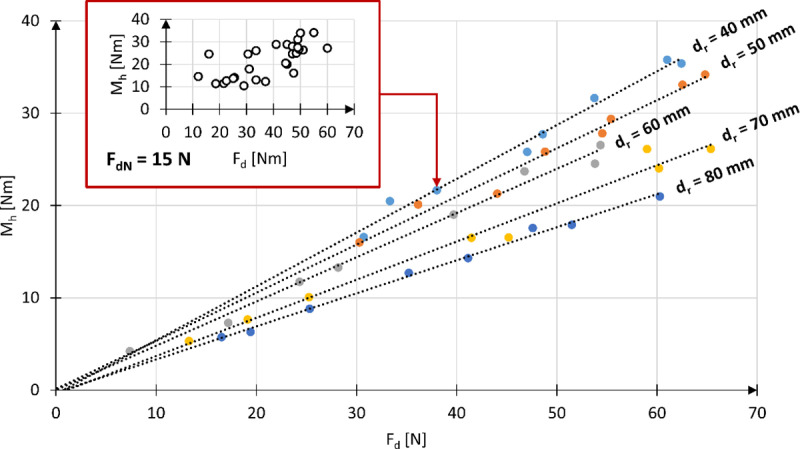
Actual characteristics of the braking moment M_h_ as a function of the pressing force F_d_ of the roller against the wheelchair wheel for various tested roller diameters. The graph includes a selected M_h_ and F_d_ curve illustrating the variation of these values over one full wheel rotation for a given constant nominal pressing force F_dN_.

The results of the averaged braking moment M_h_ and pressing force F_d_ for one full rotation of the wheelchair wheel, conducted for 8 different nominal pressing forces F_dN_ and various roller diameters d_r_, are presented in Table 3. Using the measured values of braking moment M_h_ and pressing force F_d_ during one full rotation of the wheel, the characteristics of the braking moment as a function of the pressing force of the reverse locking module roller on the wheelchair wheel were determined (Fig 13). It should be noted that each point on the resulting characteristic represents the average of 28 measurements taken at equal intervals along the circumference of the wheelchair’s drive wheel.

**Table 3:** Values of the pressing force Fd for one full rotation of the wheel at a constant nominal pressing force F_dN_, along with the corresponding values of the braking moment M_h_ for different diameters of the reverse locking module roller. The confidence interval was determined for a significance level of p = 0.05 and a sample size of n = 28.

F_dN_	d_r_ = 40 mm		d_r_ = 50 mm		d_r_ = 60 mm		d_r_ = 70 mm		d_r_ = 80 mm	
	F_d_	M_h_	F_d_	M_h_	F_d_	M_h_	F_d_	M_h_	F_d_	M_h_
	*[N]*	*[Nm]*	*[N]*	*[Nm]*	*[N]*	*[Nm]*	*[N]*	*[Nm]*	*[N]*	*[Nm]*
*5 N*	30,7 ±4,5	16,6 ±2,3	30,3 ±4,6	16,0 ±2,1	17,2 ±2,7	7,3 ±1,1	13,3 ±2,9	5,3 ±1,6	16,5 ±2,8	5,8 ±1,5
*10 N*	33,3 ±4,4	20,5 ±1,3	36,1 ±4,6	20,1 ±2,4	7,4 ±2,2	4,2 ±1,1	19,1 ±3,4	7,7 ±1,8	19,4 ±3,1	6,3 ±1,6
*15 N*	38,0 ±5,2	21,7 ±3,0	44,1 ±4,8	21,3 ±1,5	24,3 ±3,5	11,7 ±1,4	25,3 ±3,2	10,1 ±1,4	25,3 ±3,4	8,8 ±1,5
*20 N*	47,0 ±5,2	25,8 ±2,9	48,8 ±5,1	25,8 ±3,2	28,2 ±3,5	13,3 ±1,4	41,5 ±3,5	16,5 ±1,6	35,2 ±3,7	12,7 ±1,7
*25 N*	48,6 ±5,4	27,7 ±3,2	54,5 ±4,8	27,8 ±2,7	39,7 ±3,5	19,0 ±1,4	45,2 ±3,5	16,5 ±1,6	41,1 ±3,9	14,3 ±1,4
*30 N*	53,8 ±5,9	31,6 ±2,4	55,4 ±4,8	29,4 ±3,8	53,8 ±3,7	24,5 ±1,3	59,0 ±3,9	26,1 ±1,7	47,6 ±3,8	17,6 ±1,5
*35N*	62,4 ±5,9	35,4 ±3,5	62,5 ±4,9	33,1 ±2,4	46,8 ±4,0	23,7 ±1,5	60,2 ±3,8	24,0 ±1,5	51,5 ±4,1	17,9 ±1,5
*40 N*	61,0 ±7,1	35,8 ±3,4	64,8 ±4,7	34,2 ±3,2	54,4 ±4,1	26,5 ±1,4	65,4 ±3,7	26,2 ±1,5	60,3 ±4,1	21,0 ±1,4

Analysis of the actual M_h_ characteristics revealed a linear trend, which can be described by a general function [6] (Fig 13) with two parameters: ad, the slope coefficient, and b, the y-intercept. Using the actual measurement data, these parameters were determined for each of the tested reverse locking module rollers (Table 4). The values of these parameters were established for a system with a wheelchair wheel diameter of 24” and a tire pressure of p = 6 bar.

**Table 4:** Parameters of the adopted mathematical model describing the change in braking moment M_h_ as a function of the pressing force F_d_ of the roller.

d_r_	a_d_	b	R^2^
*[Nm]*	*[Nm\*	*[-]*
40 mm	0,5831	0,4362	0,98
50 mm	0,5214	0,1193	0,98
60 mm	0,4794	0,0111	0,99
70 mm	0,4136	0,4523	0,98
80 mm	0,3584	0,2622	0,99


$$M_{h}\left(F_{d}\right)=a_{d}\cdot F_{d}+b$$ (6)

Assuming that for a pressing force F_d_ = 0 N, the braking moment M_h_ generated by the friction force of the pressed roller is equal to 0 Nm, the following conclusions were drawn:

- the value of the intercept b should be 0 Nm,
- non-zero values of the intercept b in the actual characteristic (Fig 11) result from internal resistance and the measurement error of the torque sensor used.

Taking the above assumptions into account, it is possible to derive a mathematical model in which the slope coefficient ad from equation (5) is expressed as a function of the roller diameter d_r_ used in the reverse locking module (S2 Appendix B, Fig 12). A linear model was applied to describe the relationship between the slope coefficient ad and the roller diameter d_r_, as given by equation (6). The derived linear model has a coefficient of determination of R² = 0.997.


$$a_{d}\left(d_{r}\right)=-0,005572\cdot d_{r}+0,8055$$ (7)

**Fig 14:**
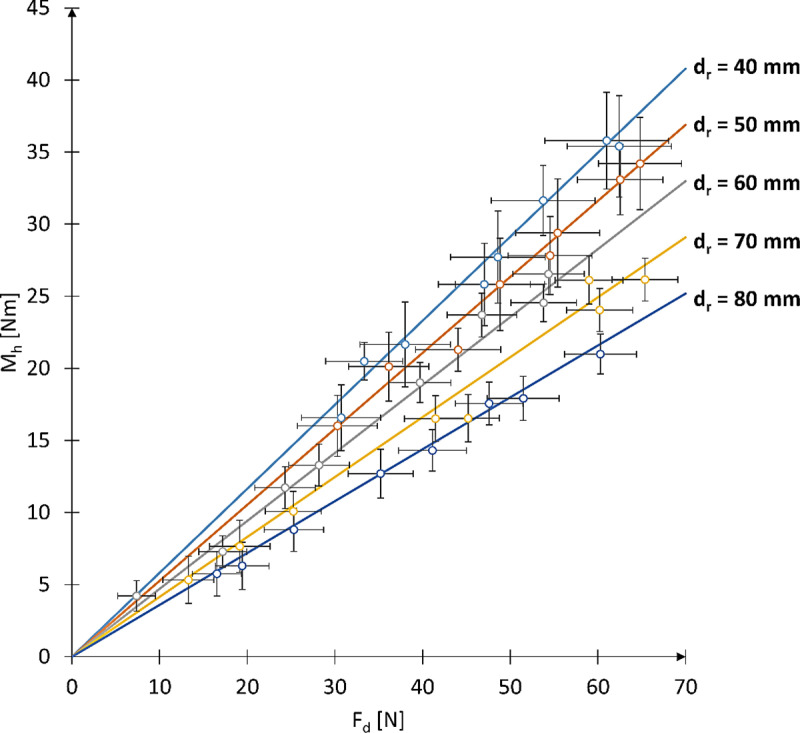
Modeled characteristic of the braking moment M_h_ as a function of the roller diameter d_r_ and the pressing force F_d_, with actual measurement points of M_h_ indicated. The actual measurement points include the confidence interval calculated for a confidence level of p = 0.05 and a sample size of n = 28.


By expressing the parameter ad from equation (5) according to equation (6), a mathematical model was derived that enables the determination of the braking moment M_h_ as a function of the diameter of the pressed roller d_r_ and the pressing force Fd [7]. The modeled M_h_ characteristic, along with the actual results, is presented in Fig 14. A comparison between the modeled and actual values showed that the developed model has an absolute error ranging from 3% to 7%.

$$M_{h}\left(d_{r},\;F_{d}\right)=\left(-0,005572\cdot d_{r}+0,8055\right)\cdot F_{d}$$ (8)

Additionally, an ANOVA analysis was conducted on the results of the experiment to evaluate the significance of the influence of the nominal pressing force F_dN_, the roller diameter d_r_, and the nominal pressing force dependent on roller diameter F_dN_(d_r_). The results of the significance analysis of these parameters on the mean pressing force F_d_ and braking moment M_h_ are presented in Tables 5 and 6. The statistical significance analysis for the parameters affecting the average pressing force F_d_ showed a significant influence of both F_dN_ (p < 0.001) and d_r_ (p < 2e-16). Moreover, there is a significant interaction between F_dN_ and d_r_ (p < 0.001), which means that the effect of F_dN_ on F_d_ varies depending on the roller diameter d_r_. For the dependent variable M_h_, the analysis also showed a significant effect of both F_dN_ (p < 0.001) and d_r_ (p < 0.001). However, there is no significant interaction between F_dN_ and d_r_ (p = 0.4867), indicating that the influence of F_dN_ on M_h_ is independent of the roller diameter d_r_. In summary, the analysis showed that both F_dN_ and d_r_ have a statistically significant effect on the values of F_d_ and M_h_. In the case of F_d_, there is also a significant interaction between these factors, meaning that the effect of F_dN_ on F_d_ differs depending on the roller diameter d_r_ used.

**Table 5:** Significance analysis of the influence of variables on the mean pressing force Fd.

Source of Variation	df	F-statistic	p-value
F_dN_	6	10.439	0.0001
d_r_	4	14.896	2e-16
F_dN_(d_r_)	24	4.001	0.0001

**Table 6:** Significance analysis of the influence of variables on the mean braking moment M_h_.

Source of Variation	df	F-statistic	p-value
F_dN_	6	6.836	0.0001
d_r_	4	5.564	0.0011
F_dN_(d_r_)	24	1.002	0.4867

### 3.3 Measurement of tire deformation under the influence of the roller’s pressing force

**Fig 15:**
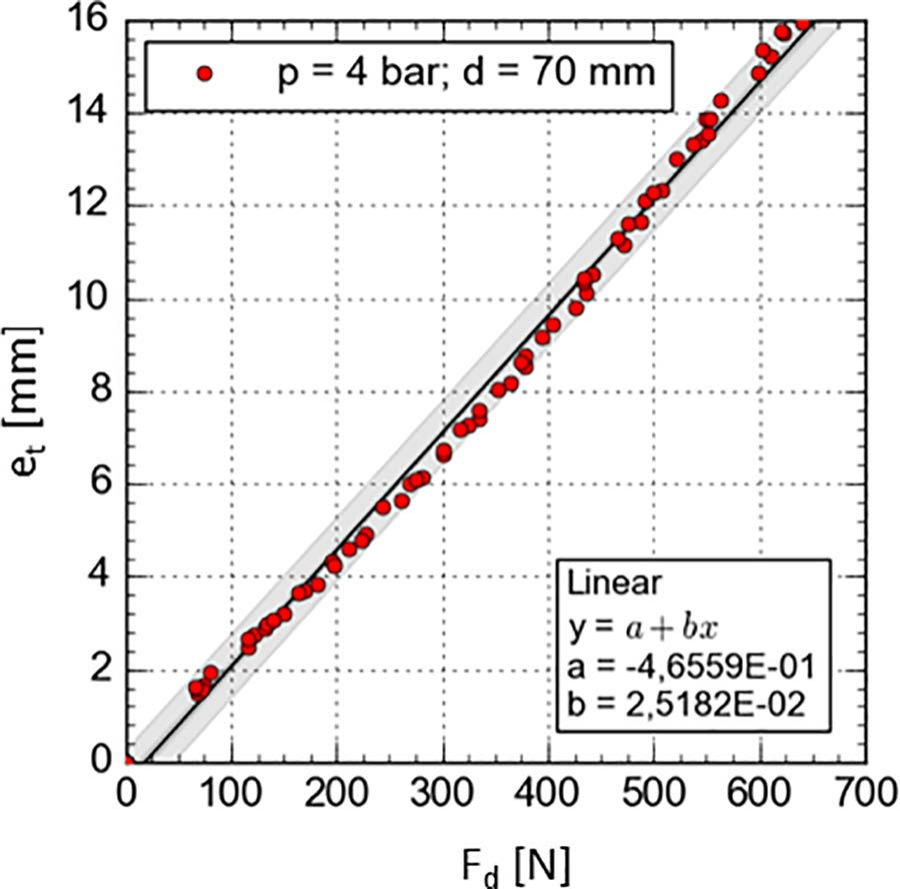
Results of tire deformation e_T_ as a function of the roller’s pressing force F_d_ for a constant tire pressure of p = 4 bar and a roller diameter of d_r_ = 70 mm.

The third experimental task focused on identifying the relationship between the pressing force Fd of the roller and the resulting tire deformation eT. This relationship is essential for building a model that indirectly estimates braking torque via deformation values. Using a custom-designed test stand, actual tire deformation characteristics e_T_ were determined as a function of the roller’s pressing force F_d_, for constant values of tire pressure and roller diameter. The deformation characteristics were established for pressure variants p ranging from 4 to 7 bar and roller diameters d_r_ from 30 to 90 mm. An example of an actual deformation characteristic for a roller with a diameter of d_r_ = 70 mm and a tire pressure of p = 4 bar is presented in Fig 15.

Analysis of the measured results showed that, for each variant of tire pressure and roller diameter d_r_ of the reverse locking module, the course of the actual deformation characteristic can be approximated using a linear function of the form [8]:


$$e_{T}\left(\;F_{d}\right)=a_{p}\cdot F_{d}+b_{p}$$ (9)

Where:

e_T_ – tire deformation expressed in millimeters (mm),
F_d_ – roller pressing force expressed in newtons (N),
a_p_ – slope coefficient dependent on the tire pressure and roller diameter,
b_p_ – intercept (constant term).

According to the adopted mathematical model, the parameters ap and bp were calculated for each tested configuration of tire pressure p and roller diameter d_r_ (Table 7). While determining the confidence intervals for these parameters, Student’s t-distribution was used, assuming a confidence level of p = 0.05 and a sample size of n > 60.

**Table 7:** Summary of the parameters of the linear tire deformation function for all variants of tire pressure p and roller diameter d_r_. Where: a_p_ – slope coefficient dependent on tire pressure and roller diameter, b_p_ – intercept, SE – standard error, U_₀_._₀₅_ – confidence interval at the 0.05 significance level, R² – coefficient of determination.

Ciśnienie opony	Średnica rolki	Parametry funkcji matematycznej [8]		Błąd standardowy	Przedział ufności	Współczynnik determinacji
p [bar]	d_r_ [mm]			SE	U_0.05_	R_2_
4	30	a_p_	0,030986	0,000301	±0,000603	0.9943
		b_p_	−0,679867	0,095371	±0,19077	
	40	a_p_	0,029400	0,000271	±0,000542	0.9948
			b_p_	−0,672310	0,091995	±0,183895
	50	a_p_	0,027180	0,000237	±0,00048	0.9958
			b_p_	−0,541380	0,082353	±0,16497
	60	a_p_	0,026614	0,000257	±0,000514	0,9972
			b_p_	−0,636631	0,098605	±0,197239
	70	a_p_	0,025182	0,000219	±0,000436	0.9950
			b_p_	−0,465591	0,080624	±0,160926
	80	a_p_	0,024326	0,000242	±0,000482	0.9934
			b_p_	−0,595171	0,100212	±0,200024
	90	a_p_	0,023107	0,000174	±0,000348	0.9964
			b_p_	−0,392779	0,073376	±0,146586
5	30	a_p_	0,027015	0,000229	±0,000458	0.9954
		b_p_	−0,486434	0,081623	±0,16306	
	40	a_p_	0,024413	0,024413	±0,000415	0.9951
			b_p_	−0,466126	0,083045	±0,165714
	50	a_p_	0,023146	0,000170	±0,00034	0.9968
			b_p_	−0,387345	0,071667	±0,143355
	60	a_p_	0,022749	0,000176	±0,00035	0.9959
			b_p_	−0,424942	0,075430	±0,15056
	70	a_p_	0,021517	0,000148	±0,0003	0.9967
			b_p_	−0,380604	0,069041	±0,13781
	80	a_p_	0,020669	0,000127	±0,00025	0.9975
			b_p_	−0,308047	0,060334	±0,12043
	90	a_p_	0,019701	0,000127	±0,00025	0.9972
			b_p_	−0,286556	0,059647	±0,11906
6	30	a_p_	0,022087	0,000186	0,000372	0.9955
		b_p_	−0,346486	0,081711	0,163237	
	40	a_p_	0,020664	0,000154	0,000308	0.9962
			b_p_	−0,299676	0,071561	0,142797
	50	a_p_	0,019800	0,000244	0,000486	0.9900
			b_p_	−0,343569	0,115835	0,231208
	60	a_p_	0,019620	0,000164	0,000327	0.9953
			b_p_	−0,34541	0,088698	0,176994
	70	a_p_	0,018146	0,000149	0,000297	0.9955
			b_p_	−0,193485	0,074548	0,148799
	80	a_p_	0,016629	0,000101	0,000203	0.9975
			b_p_	−0,129595	0,054250	0,108283
	90	a_p_	0,015888	0,000112	0,000224	0.9967
			b_p_	−0,089650	0,060406	0,120571
7	30	a_p_	0,019412	0,000196	0,000391	0.9935
		b_p_	−0,218262	0,105328	0,21041	
	40	a_p_	0,018449	0,000206	0,00041	0.9916
			b_p_	−0,119278	0,103798	0,207126
	50	a_p_	0,017394	0,000200	0,0004	0.9922
			b_p_	−0,111459	0,107619	0,215271
	60	a_p_	0,017218	0,000187	0,000372	0.9922
			b_p_	−0,202610	0,101962	0,203517
	70	a_p_	0,016490	0,000137	0,000272	0.9954
			b_p_	−0,099800	0,077514	0,154718
	80	a_p_	0,015678	0,000131	0,000261	0.9953
			b_p_	−0,166544	0,070725	0,141168
	90	a_p_	0,015143	0,000108	0,000215	0.9966
			b_p_	−0,088464	0,058569	0,116906

Under ideal conditions unaffected by measurement error, the intercept b_p_ should be equal to 0, since for a pressing force F_d_ = 0 N, the tire deformation e_T_ should also be 0 mm. Therefore, in the process of formulating the mathematical model, a zero value for the intercept b_p_ was assumed. By grouping the linear function parameters according to the tire pressure p, it was observed that the slope coefficient a_p_ for a given constant tire pressure is dependent on the diameter of the pressed roller d_r_. To establish this dependency, a statistical analysis was conducted to determine the trend line of the ap parameter as a function of roller diameter d_r_ for a constant tire pressure p (S2 Appendix B, Fig 15).

Taking into account the above assumptions, the results of experimental studies, and the statistical analysis, it is possible for a constant tire pressure p to transform the linear function describing the actual course of the tire deformation characteristic into a mathematical model dependent on the roller diameter d_r_ and the pressing force F_d_ (equation 9). In this model, the parameters k₁,_p_ and k₂,_p_, determined experimentally, define the function’s dependency on the tire pressure value. The values of the parameters k₁,_p_ and k₂,_p_ are presented in the table below (Table 8) and are assigned to specific tire pressure values used in the wheelchair wheel.

**Table 8:** Summary of constants k₁,_p_ and k₂,_p_ dependent on tire pressure.

Tire Pressure	Constants in Tire Pressure Model (Equation 9)		Standard Error	Confidence Interval	Coefficient of Determination
p [bar]			SE	U₀.₀₅	R²
4	k_1,4 bar_	0,034353	0,000539	0,0013855	0.9781
	k_2, 4 bar_	−0,000128	0,000009	0,000022	
5	k_1,5 bar_	0,029400	0,000719	0,001847	0.9502
	k_2,5 bar_	−0,000111	0,000011	0,000029	
6	k_1,6 bar_	0,025045	0,000450	0,001157	0.9759
	k_2,6 bar_	−0,000101	0,000007	0,000018	
7	k_1,7 bar_	0,021238	0,000267	0,000687	0.9815
	k_2,7 bar_	−0,000069	0,000004	0,000011	


$$e_{T}\left(\;F_{d},d_{r}\right)=\left(k_{1,p}+k_{2,p}d_{r}\right)\cdot F_{d}$$ (10)

Where:

e_T_ – tire deformation,
F_d_ – pressing force of the reverse locking module roller,
d_r_ – diameter of the reverse locking module roller,
k₁,_p_ – pressure-dependent constant (intercept term),
k₂,_p_ – pressure-dependent slope coefficient,
p – tire pressure.

**Fig 16:**
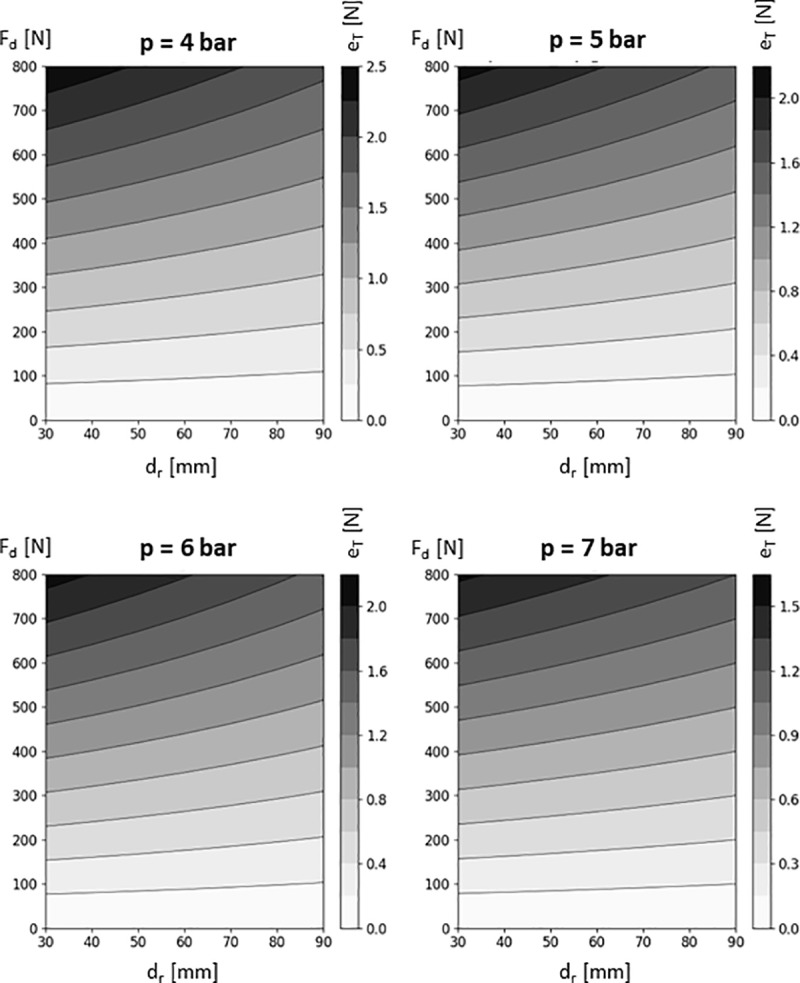
Graph of tire deformation e_T_ as a function of the roller pressing force Fd and roller diameter d_r_, developed based on the derived mathematical model [9].

The tire deformations determined based on the mathematical model [9], dependent on the roller diameter d_r_ and the pressing force F_d_, are presented in Fig 16.

By comparing the developed mathematical models with the actual characteristics of the phenomenon obtained from the experiment, the absolute error was calculated (Table 10). Analysis of the results showed that the error between the actual characteristic and the one determined using the mathematical model does not exceed 3%. The established characteristics were prepared for four tire pressure values, ranging from the minimum pressure that allows safe wheelchair operation without damage (4 bar), up to the maximum pressure achievable with household air compressors and those available at fuel stations (8 bar). The analysis of the absolute error of the developed model indicated that the smallest error was obtained for a tire pressure of 7 bar. This is a favorable result, as high-pressure tires with a working pressure range of 6–10 bar are most commonly used, and household compressors typically generate a maximum pressure in the range of 7–8 bar (Table 9).

**Table 9:** Summary of the absolute error of the mathematical model in relation to the actual tire deformation characteristics e_T_, which depend on the pressing force Fd and the roller diameter d_r_ of the reverse locking module. Where: p – tire pressure, d_r_ – diameter of the reverse locking module roller.

p	d_r_							Avg. error
30 mm	40 mm	50 mm	60 mm	70 mm	80 mm	90 mm	
4 bar	1,53%	0,57%	2,84%	0,22%	0,84%	0,88%	1,19%	1,15 ± 1,03%
5 bar	3,50%	2,24%	3,04%	0,04%	0,53%	0,72%	1,48%	1,65 ± 1,64%
6 bar	0,33%	1,65%	0,98%	3,24%	0,94%	2,02%	0,42%	1,37 ± 1,27%
7 bar	1,26%	0,16%	2,27%	0,70%	0,50%	0,26%	0,76%	0,84 ± 0,90%

### 3.4 Braking moment model as a function of tire deformation

Based on the results from Sections 3.1 to 3.3, this section presents the analytical objective of the study: the derivation of a generalized mathematical model that correlates tire deformation with braking torque. This model enables estimation of the braking moment M_h_ without direct measurement of pressing force. The conducted experimental studies made it possible to determine mathematical models for the braking moment M_h_ (equation 10) and tire deformation e_T_ [11] as functions of the pressing force Fd and the roller diameter d_r_ applied to the wheelchair wheel. To achieve the research objective, these mathematical functions must be combined by expressing M_h_ as a function of e_T_.


$$M_{h}\left(d_{r},\;F_{d}\right)=\left(-0,005572\cdot d_{r}+0,8055\right)\cdot F_{d}$$ (11)

$$e_{T}\left(\;F_{d},d_{r}\right)=\left(k_{1,p}+k_{2,p}d_{r}\right)\cdot F_{d}$$ (12)

By rearranging equation (11) to solve for the pressing force F_d_ and substituting it into equation (10), the desired mathematical model of the braking moment M_h_ as a function of tire deformation e_T_ and the roller diameter d_r_ of the reverse locking module was derived [12]. Additionally, the model incorporates the pressure-dependent constants k₁,_p_ and k₂,_p_, which were determined in the previously described experiment.


$$M_{h}=\frac{\left(0,8055-0,005572d_{r}\right)\cdot e_{T}}{k_{1,p}+k_{2,p}d_{r}}$$ (13)

To implement the model under real-world boundary conditions resulting from wheelchair operation, it was transformed to specifically describe the interaction between the roller and a wheelchair tire with an internal pressure of 6 bar [13]. This pressure represents the minimum operating value for high-pressure wheelchair tires, while also corresponding to the upper limit typically achievable using household air compressors commonly used by wheelchair users.


$$M_{h}=\frac{\left(0,8055-0,005572d_{r}\right)\cdot e_{T}}{0,025045-0,000101d_{r}}$$ (14)

Where:

M_h_ – braking moment generated by the reverse locking module roller pressed against a 24“ × 1” tire with an internal pressure of 6 bar,
d_r_ – diameter of the reverse locking module roller,
e_T_ – tire deformation resulting from the pressing force of the reverse locking module roller.

**Fig 17:**
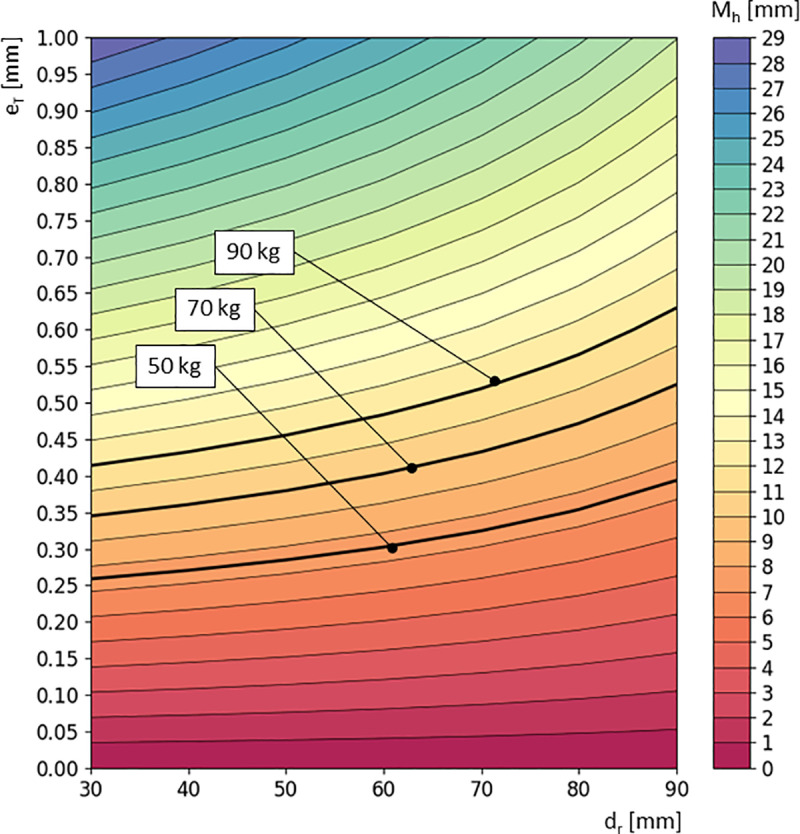
Graph of the braking moment M_h_ as a function of tire deformation e_T_ and the diameter of the pressed roller d_r_ of the reverse locking module, for a tire pressure of p = 6 bar. The graph includes the M_h_ curves corresponding to a wheelchair inclination of 4.56° for user body masses of 90 kg, 70 kg, and 50 kg.

The above mathematical model is illustrated in the graph (Fig 17), indicating the minimum braking moment M_h_ that must be generated in order to stop a wheelchair with a mass of 17.7 kg on an incline with a slope of 4.56°. This wheelchair is intended for users with body masses of 50 kg, 70 kg, and 90 kg.

## 4. Discussion

The studies aimed at developing a model describing the generated braking moment M_h_ as a function of tire deformation e_T_ enable the adjustment of friction-coupled components with the wheelchair’s drive wheel without the need for specialized measurement equipment, such as force sensors. As a result, users can calibrate the parking brake [36,37] or assistive modules for ascending inclines—such as anti-rollback devices [38,39]. The research and model presented in this work are applicable to all wheelchairs equipped with 24“ × 1” wheels. This constraint in the experimental setup was intentional, as drive wheels with these geometric characteristics are the most common in manual wheelchairs used by adults [40]. The developed mathematical model of M_h_ as a function of e_T_ is also limited by the range of tire pressures for which it was validated. In its presented form, the model is valid for pressures ranging from 4 to 7 bar. This range is consistent with previous studies by other researchers [41,42] and with the recommendations of the tire manufacturer, who advises a nominal pressure of 6 bar. It is worth noting that 6 bar also represents the maximum pressure that can typically be achieved using household air compressors.

Using the model variant for a tire pressure of 6 bar, a detailed analysis was performed on the influence of the roller diameter d_r_ and the tire deformation e_T_ on the value of the generated braking moment M_h_. It was found that both variables are statistically significant, with the greatest significance shown by the tire deformation e_T_ (p = 8.761940e-95). A secondary, yet still highly statistically significant influence was observed for the roller diameter d_r_ (p = 5.334779e-32). For the tested range of roller diameters, to brake a wheelchair with a 90 kg user on a slope of 4.6°, it is sufficient to press the roller so as to produce a tire deformation within the range of 0.41 mm to 0.63 mm. Based on this, it was concluded that increasing the roller diameter d_r_ by 60 mm requires an increase in pressing force that causes a rise in deformation of only 0.22 mm. This increase in deformation is enough to maintain a constant braking moment M_h_ of 12 Nm. On the other hand, assuming a constant roller diameter d_r_, for example 70 mm, in order to generate a braking moment M_h_ of 7.5 Nm (a user with a mass of 50 kg on a 4.56° slope), a deformation e_T_ of 0.32 mm must be induced. In the case of M_h_ equal to 12 Nm (a 90 kg user on a 4.56° slope), the tire must be deformed by e_T_ = 0.52 mm, which constitutes a difference of 0.2 mm compared to M_h_ = 7.5 Nm. Referring this analysis to the research problem of establishing adjustment guidelines for the wheelchair anti-rollback module in order to ensure correct operation of the module on standardized ramps, the deformation value e_T_ should fall within the range of 0.26 mm to 0.63 mm.

The above deformation range e_T_ was calculated using the braking moment M_h_ value determined experimentally based on the measurement of the sliding force F_Z_ acting on a wheelchair positioned on an incline. The experiment showed that the user’s mass has the greatest influence on the value of the sliding force F_Z_. An increase in user mass from 50 kg to 90 kg results in a 57% increase in the maximum value of the sliding force (e.g., from 107.58 N to 175.33 N at 7 bar pressure and a 10° slope). This phenomenon can be explained by the fact that a greater mass increases the gravitational force acting on the user. This result is consistent with the findings of other authors, who have indicated the significance of the human–wheelchair system mass on the rolling resistance force resulting from terrain inclination [43–45].

Regarding the influence of tire pressure p in the wheelchair wheel, it was found to be of secondary importance in the context of the sliding force F_Z_. Although the differences in F_Z_ values between 3 and 7 bar are noticeable, they are smaller compared to those caused by changes in user mass. The highest percentage difference Δ_p7−3_ was observed for small slope angles (α ≤ 3.5°), where it ranged from 19% to 36%. This phenomenon can be explained by the fact that at small inclination angles, rolling resistance caused by tire deformation [46–48] has a significant impact on the sliding force. However, as the slope angle increases, the influence of pressure stabilizes at around 5.4%, indicating its lesser importance at steeper inclines.

In the studies on braking moment M_h_ as a function of the roller’s pressing force F_d_ against the drive wheel of a wheelchair, a key aspect was the determination of F_d_ as the average value resulting from one full rotation of the wheelchair wheel. This necessity arose due to geometric imperfections of the wheelchair wheel. This is confirmed by the curvature analysis of the wheel through measurements of the variation in pressing force. It showed that at the point on the wheel with the smallest radius, the Fd value was 6.9 N ± 3.5 N for a fixed roller position relative to the wheel, whereas at the point with the largest radius, it reached 53.9 N ± 6.0 N. The importance of accounting for wheel curvature is also supported by the work of other researchers analyzing the effect of wheel curvature on vehicle suspension systems through variations in kinematic excitations [49,50].

**Fig 18:**
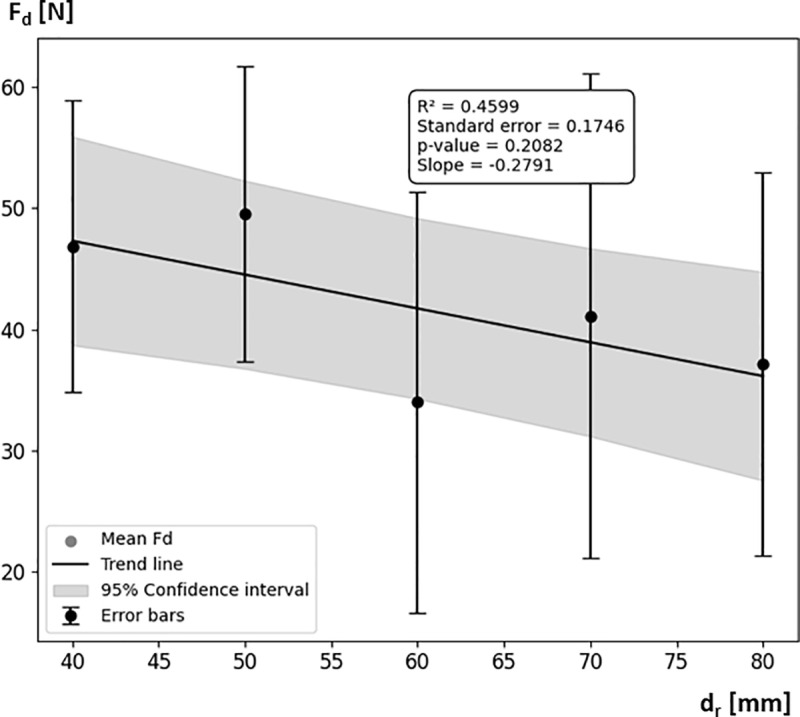
Graph showing the influence of the roller diameter d_r_, pressed against the wheelchair wheel, on the average pressing force F_d_ determined across the entire tested range of nominal pressing force F_dN_.

**Fig 19:**
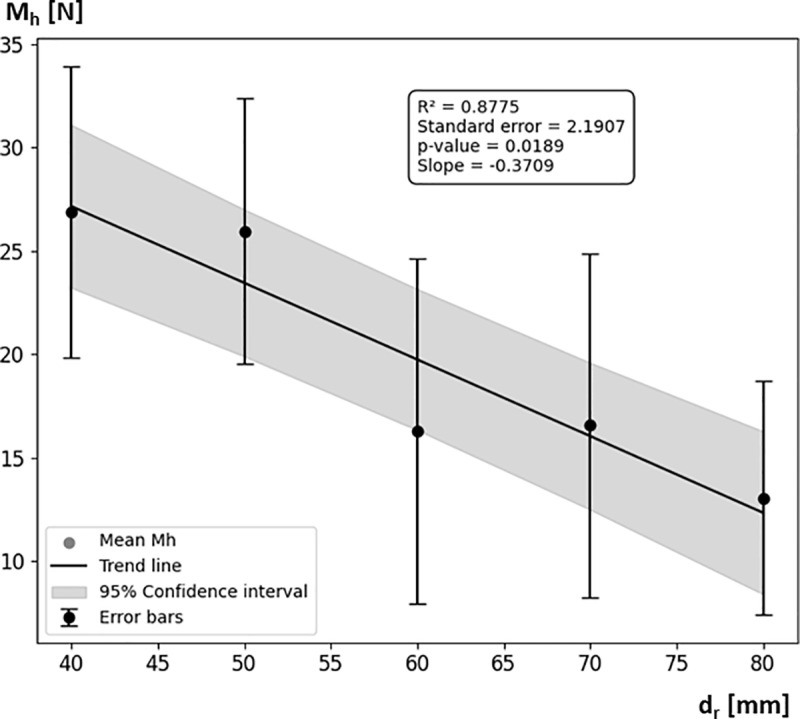
Graph of the influence of the roller diameter d_r_, pressed against the wheelchair wheel, on the average braking moment M_h_ determined for the entire tested range of nominal pressing force F_dN_.

The analysis of the results indicates varying values of the slope coefficient ad for different roller diameters d_r_ (Table 3). For example, for a roller with a diameter of 40 mm, the coefficient is 0.5831, which means that for every 1 N increase in pressing force, the braking moment increases by approximately 0.5831 Nm. These values decrease with increasing roller diameter, which is reflected in equation (6). This trend suggests that larger rollers generate smaller braking moments M_h_ in response to the same nominal pressing force F_dN_. This is confirmed by the analysis of the pressing force F_d_ and braking moment M_h_ values for rollers with diameters of 40 mm, 50 mm, 60 mm, 70 mm, and 80 mm (Figs 18, 19). The analysis showed that as the roller diameter d_r_ increases, the actual pressing force F_d_—influenced by the nominal pressing force F_dN_ and the out-of-roundness of the wheelchair wheel—decreases. For d_r_ = 40 mm, the average F_d_ (mean value across the tested range of F_dN_) was measured at 46.85 ± 10.07 N, while for d_r_ = 80 mm, the average Fd was 37.11 ± 12.46 N. Analyzing the trend of the mean F_d_ value as a function of roller diameter d_r_, a decreasing tendency is observed, expressed by a linear function with a slope of −15.6°, indicating a significant downward trend. The decrease in F_d_ value caused by the increase in d_r_ directly translates into a reduction in the generated braking moment M_h_.

The analysis performed on the influence of roller diameter d_r_ on the braking moment M_h_ showed that increasing the roller diameter d_r_ also results in a decrease in M_h_ value. For d_r_ = 40 mm, the average M_h_ (mean value across the entire tested range of nominal pressing force F_dN_) was 26.89 ± 5.91 Nm, whereas for d_r_ = 80 mm, it was 13.05 ± 4.75 Nm. This represents a 51.5% decrease in M_h_, while the corresponding decrease in F_d_ was 20.8%. Based on this, it can be concluded that the reduction in M_h_ with increasing roller diameter d_r_ is caused not only by the decrease in pressing force F_d_, but also by reduced deformation of the wheelchair tire. This, in turn, leads to a reduction in sliding friction resistance [51] which relies on tire deformation [52–54].

Larger roller diameters d_r_ contribute to a reduction in tire deformation. For example, for a diameter of 30 mm and a pressure of 4 bar, a deformation e_T_ of 18 mm was obtained at a pressing force F_d_ of 600 N, whereas for d_r_ = 90 mm, the same deformation required a pressing force of 800 N. This finding highlights the importance of selecting an appropriate roller diameter in the design of a wheelchair’s anti-rollback module. The derived mathematical models accurately reflect the actual F_d_(e_T_) characteristics. The absolute error analysis showed that, for a tire pressure of 7 bar, the error did not exceed 0.84%. Such a low error indicates high precision in the obtained results and the effectiveness of the mathematical model in predicting tire deformation under real conditions. These findings may be particularly valuable for engineers and designers, who can use this data to optimize wheelchair construction, enhancing both user comfort and safety.

To confirm the achievement of the research objectives, each of the conducted experiments provided concrete numerical data that were directly utilized in the development of the analytical model. The first experimental task involved determining the sliding moment of the wheelchair as a function of slope inclination and allowed the estimation of the minimum required braking torque. For a slope angle of 6 degrees and a wheelchair mass of 80 kilograms, the calculated sliding torque was approximately 7.13 newton-meters. In the second task, the relationship between roller pressing force and braking torque was established. For a roller diameter of 50 millimeters and a pressing force of 80 newtons, the resulting braking torque reached approximately 8.25 newton-meters, which exceeds the required minimum. The third experimental task focused on the correlation between roller force and tire deformation. For a tire pressure of 2.0 bar and a pressing force of 60 newtons, the measured tire deformation was approximately 0.92 millimeters. These three empirical relationships, namely braking torque as a function of pressing force, tire deformation as a function of pressing force, and sliding torque as a function of slope angle, were mathematically combined to derive the final model that describes braking torque as a function of tire deformation. This model represents the core outcome of the study and confirms the validity of the research hypothesis by providing a simplified method for regulating braking torque based solely on tire deformation measurements, without the use of force sensors.

## 5. Conclusion

In summary, the conducted research provides valuable insights into the influence of pressing force and roller diameter on the braking moment in wheelchairs. The obtained results highlight the need for further studies to optimize wheelchair designs and may serve as a foundation for the development of more advanced mathematical models. It will also be crucial to consider potential measurement errors and their impact on the final experimental outcomes. The research focused on modeling the braking moment (M_h_) as a function of tire deformation enables the adjustment of wheelchair brakes without the need for specialized equipment. The user can adjust the brake using only a caliper. The model applies to wheelchairs equipped with 24“ × 1” wheels, which are the most commonly used in manual wheelchairs. The pressure range for which the model has been validated is 4–7 bar, with 6 bar recommended.

The analysis showed that both the diameter of the roller pressed against the tire and the tire deformation have a significant impact on M_h_, with deformation having a greater influence. To brake a wheelchair with a user weighing 90 kg on a 4.56° incline, a tire deformation in the range of 0.41 mm to 0.63 mm is sufficient. Increasing the roller diameter by 60 mm requires only a small increase in deformation about 0.22 mm. It was also determined that the user’s mass has a key influence on the sliding force (F_Z_) of the wheelchair on an incline, while changes in tire pressure play a lesser role.

The studies showed that for larger roller diameters (d_r_), the braking moment M_h_ decreases, which results from both reduced pressing force (F_d_) and smaller tire deformation. It was also determined that selecting the appropriate roller diameter is crucial when designing the anti-rollback module. Ultimately, the developed mathematical models exhibit high precision, which may be particularly important for engineers and wheelchair designers, enabling better construction and improved user comfort.

## Supporting information

S1 Appendix A Results of the sliding force experiment. Contains graphs and datasets presenting the relationship between ramp inclination, user mass, and tire pressure with the sliding force FZ. Related to Figure 9.
(PDF)

S2 Appendix B Supplementary equations and regression models. Includes figures and mathematical models supporting the analysis of braking torque as a function of roller pressing force and tire deformation. Related to Figures 12, 14, 15, and 17.
(PDF)

S1 Data DATA
(XLSX)

## References

[1] Sakakibara BM, Miller WC, Eng JJ, Routhier F, Backman CL. Health, personal, and environmental predictors of wheelchair-use confidence in adult wheelchair users. Phys Ther. 2015;95(10):1365–73. doi: 10.2522/ptj.20140537 25953595 PMC4595810

[2] Sakakibara BM, Miller WC, Eng JJ, Backman CL, Routhier F. Influences of wheelchair-related efficacy on life-space mobility in adults who use a wheelchair and live in the community. Phys Ther. 2014;94(11):1604–13. doi: 10.2522/ptj.20140113 24925076 PMC4221814

[3] Smith EM, Sakakibara BM, Miller WC. A review of factors influencing participation in social and community activities for wheelchair users. Disabil Rehabil Assist Technol. 2016;11(5):361–74. doi: 10.3109/17483107.2014.989420 25472004 PMC4581875

[4] Dallmeijer AJ, van der Woude LH, Veeger HE, Hollander AP. Effectiveness of force application in manual wheelchair propulsion in persons with spinal cord injuries. Am J Phys Med Rehabil. 1998;77(3):213–21. doi: 10.1097/00002060-199805000-00006 9635556

[5] Rifai Sarraj A, Massarelli R, Rigal F, Moussa E, Jacob C, Fazah A, et al. Evaluation of a wheelchair prototype with non-conventional, manual propulsion. Ann Phys Rehabil Med. 2010;53(2):105–17. doi: 10.1016/j.rehab.2009.12.001 20060796

[6] Kim CS, Lee D, Lee J, Kwon S, Chung MK. Effects of ramp slope and height on usability and physiology during wheelchair driving. Proc Hum Factors Ergon Soc Annu Meet. 2010;54(9):698–702. doi: 10.1177/154193121005400903

[7] Miyawaki K, Sasaki M, Iwami T, Obinata G, Shimada Y. Evaluation of dynamics of pushing a wheelchair up or down a slope. JSDD. 2012;6(4):525–36. doi: 10.1299/jsdd.6.525

[8] Qi L, Zhang L, Lin X-B, Ferguson-Pell M. Wheelchair propulsion fatigue thresholds in electromyographic and ventilatory testing. Spinal Cord. 2020;58(10):1104–11. doi: 10.1038/s41393-020-0470-2 32367012

[9] Rice I, Impink B, Niyonkuru C, Boninger M. Manual wheelchair stroke characteristics during an extended period of propulsion. Spinal Cord. 2009;47(5):413–7. doi: 10.1038/sc.2008.139 19002155

[10] Rodgers MM, Gayle GW, Figoni SF, Kobayashi M, Lieh J, Glaser RM. Biomechanics of wheelchair propulsion during fatigue. Archives of Physical Medicine and Rehabilitation. 1994;75(1):85–93. doi: 10.1016/0003-9993(94)90343-3 8291970

[11] Lawn M, Takeda T. Design of a robotic-hybrid wheelchair for operation in barrier present environments. In: Proceedings of the 20th Annual International Conference of the IEEE Engineering in Medicine and Biology Society, 1998. 2678–81.

[12] Podobnik J, Rejc J, Slajpah S, Munih M, Mihelj M. All-Terrain wheelchair: increasing personal mobility with a powered wheel-track hybrid wheelchair. IEEE Robot Automat Mag. 2017;24(4):26–36. doi: 10.1109/mra.2017.2746182

[13] Wieczorek B, Warguła Ł, Kukla M. Influence of a hybrid manual–electric wheelchair propulsion system on the user’s muscular effort. Acta Mechanica et Automatica. 2023;17(1):28–34. doi: 10.2478/ama-2023-0003

[14] Wieczorek B, Warguła Ł, Rybarczyk D. Impact of a hybrid assisted wheelchair propulsion system on motion kinematics during climbing up a slope. Appl Sci. 2020;10(3):1025. doi: 10.3390/app10031025

[15] Deems-Dluhy SL, Jayaraman C, Green S, Albert MV, Jayaraman A. Evaluating the functionality and usability of two novel wheelchair anti-rollback devices for ramp ascent in manual wheelchair users with spinal cord injury. PM R. 2017;9(5):483–93. doi: 10.1016/j.pmrj.2016.09.007 27664403

[16] Wieczorek B, Kukla M, Warguła Ł, Giedrowicz M, Rybarczyk D. Evaluation of anti-rollback systems in manual wheelchairs: muscular activity and upper limb kinematics during propulsion. Sci Rep. 2022;12(1):19061. doi: 10.1038/s41598-022-21806-z 36351954 PMC9646883

[17] Wieczorek B, Kukla M, Rybarczyk D, Warguła Ł. Evaluation of the biomechanical parameters of human-wheelchair systems during ramp climbing with the use of a manual wheelchair with anti-rollback devices. Appl Sci. 2020;10(23):8757. doi: 10.3390/app10238757

[18] Tanaka Y.; Murata S. Influence of tire deformation on sound pressure level inside a tire. INTER-NOISE NOISE-CON Congr. Conf. Proc. 2016;253:3542–3547.

[19] Li Y, Zuo S, Lei L, Yang X, Wu X. Analysis of impact factors of tire wear. J Vib Control. 2011;18(6):833–40. doi: 10.1177/1077546311411756

[20] Wu B-F, Chang P-J, Chen Y-S, Huang C-W. An Intelligent wheelchair anti-lock braking system design with friction coefficient estimation. IEEE Access. 2018;6:73686–701. doi: 10.1109/access.2018.2884658

[21] Fancher P, Bernard J, Clover C, Winkler C. Representing truck tire characteristics in simulations of braking and braking-in-a-Turn maneuvers. Veh Syst Dyn. 1997;27(sup001):207–20. doi: 10.1080/00423119708969655

[22] Gim G, Choi Y, Kim S. A semi-physical tire model for a vehicle dynamics analysis of handling and braking. Veh Syst Dyn. 2007;45(sup1):169–90. doi: 10.1080/00423110701723799

[23] Koo S-L, Tan H-S. Tire dynamic deflection and its impact on vehicle longitudinal dynamics and control. IEEE/ASME Trans Mechatron. 2007;12(6):623–31. doi: 10.1109/tmech.2007.910073

[24] Yang S, Li S, Lu Y. Dynamics of vehicle-pavement coupled system based on a revised flexible roller contact tire model. Sci China Ser E-Technol Sci. 2009;52(3):721–30. doi: 10.1007/s11431-009-0053-0

[25] Eckert JJ, Bertoti E, Costa E dos S, Santiciolli FM, Yamashita RY, Silva LC de A e, et al. Experimental Evaluation of Rotational Inertia and Tire Rolling Resistance for a Twin Roller Chassis Dynamometer; SAE International: Warrendale, PA, 2017.

[26] Fedotov AI, Krivtsov SN, Yankov OS. Circulation of Power During Braking of Tyre of Vehicle Wheel On Support Rollers of He Diagnostic Stand.; Atlantis Press, May 2018; pp. 147–151.

[27] Lourenço MA de M, Eckert JJ, Silva FL, Santiciolli FM, Silva LCA. Vehicle and twin-roller chassis dynamometer model considering slip tire interactions. Mech. Based Des. Struct. Mach. 2022;51(11):6166–83. doi: 10.1080/15397734.2022.2038199

[28] Kirby RL, Ashton BD, Ackroyd-Stolarz SA, MacLeod DA. Adding loads to occupied wheelchairs: effect on static rear and forward stability. Arch Phys Med Rehabil. 1996;77(2):183–6. doi: 10.1016/s0003-9993(96)90165-3 8607744

[29] Abeels PFJ. Tire deflection and contact studies. J Terramechanics. 1976;13(3):183–96. doi: 10.1016/0022-4898(76)90005-7

[30] Hambleton JP, Drescher A. Modeling test rolling on cohesive subgrades.

[31] Nakashima H, Wong JY. A three-dimensional tire model by the finite element method. J Terramechanics. 1993;30(1):21–34. doi: 10.1016/0022-4898(93)90028-v

[32] Nega A, Gedafa D. Influence of Tire Footprint Contact Area and Pressure Distribution on Flexible Pavement. In: Airfield and Highway Pavements 2021, 2021. 1–12. doi: 10.1061/9780784483503.001

[33] Jang H-S, Zhang Q-Z, Kang S-S, Jang B-A. Determination of the basic friction angle of rock surfaces by tilt tests. Rock Mech Rock Eng. 2017;51(4):989–1004. doi: 10.1007/s00603-017-1388-7

[34] Garshelis I.J. Torque and Power Measurement. In Mechanical Variables Measurement - Solid, Fluid, and Thermal; CRC Press, 1999 ISBN 978-1-003-41821-4.

[35] Warguła Ł, Lijewski P, Kukla M. Influence of non-commercial fuel supply systems on small engine SI exhaust emissions in relation to European approval regulations. Environ Sci Pollut Res. 2022;29(37):55928–43. doi: 10.1007/s11356-022-19687-w 35325380

[36] Cicenia EF, Hoberman M, Sampson OC. Maintenance and minor repairs of the wheelchair. Am J Phys Med. 1956;35(4):206–17. 13354736

[37] Wu B-F, Chang P-J, Chen Y-S, Huang C-W. An intelligent wheelchair anti-lock braking system design with friction coefficient estimation. IEEE Access. 2018;6:73686–701. doi: 10.1109/access.2018.2884658

[38] Deems-Dluhy SL, Jayaraman C, Green S, Albert MV, Jayaraman A. Evaluating the functionality and usability of two novel wheelchair anti-rollback devices for ramp ascent in manual wheelchair users with spinal cord injury. PM R. 2017;9(5):483–93. doi: 10.1016/j.pmrj.2016.09.007 27664403

[39] Wieczorek B, Kukla M, Rybarczyk D, Warguła Ł. Evaluation of the biomechanical parameters of human-wheelchair systems during ramp climbing with the use of a manual wheelchair with anti-rollback devices. Appl Sci. 2020;10(23):8757. doi: 10.3390/app10238757

[40] Mason BS, van der Woude LHV, Goosey-Tolfrey VL. The ergonomics of wheelchair configuration for optimal performance in the wheelchair court sports. Sports Med. 2013;43(1):23–38. doi: 10.1007/s40279-012-0005-x 23315754

[41] de Groot S, Vegter RJK, van der Woude LHV. Effect of wheelchair mass, tire type and tire pressure on physical strain and wheelchair propulsion technique. Med Eng Phys. 2013;35(10):1476–82. doi: 10.1016/j.medengphy.2013.03.019 23642660

[42] Sawatzky BJ, Kim WO, Denison I. The ergonomics of different tyres and tyre pressure during wheelchair propulsion. Ergonomics. 2004;47(14):1475–83. doi: 10.1080/00140130412331290862 15697064

[43] Ackermann M, Leonardi F, Costa HR, Fleury AT. Modeling and optimal control formulation for manual wheelchair locomotion: The influence of mass and slope on performance. In: Proceedings of the 5th IEEE RAS/EMBS International Conference on Biomedical Robotics and Biomechatronics, 2014. 1079–84.

[44] Hurd WJ, Morrow MMB, Kaufman KR, An K-N. Influence of varying level terrain on wheelchair propulsion biomechanics. Am J Phys Med Rehabil. 2008;87(12):984–91. doi: 10.1097/PHM.0b013e31818a52cc 18824889 PMC3899823

[45] Lee K, Lee C-H, Hwang S, Choi J, Bang Y. Power-Assisted wheelchair with gravity and friction compensation. IEEE Trans Ind Electron. 2016;63(4):2203–11. doi: 10.1109/tie.2016.2514357

[46] Ding Y, Wang H, Qian J, Zhou H. Evaluation of tire rolling resistance from tire-deformable pavement interaction modeling. J Transp Eng, Part B: Pavements. 2021;147(3). doi: 10.1061/jpeodx.0000295

[47] Hall DE, Moreland JC. Fundamentals of rolling resistance. Rubber Chem Technol. 2001;74(3):525–39. doi: 10.5254/1.3547650

[48] Xiong Y, Tuononen A. Rolling deformation of truck tires: measurement and analysis using a tire sensing approach. J Terramechanics. 2015;61:33–42. doi: 10.1016/j.jterra.2015.07.004

[49] Ali Rezvani M, Asadi Lari A. The effect of kinematic oscillations on harmonic wheel flange wear of rail vehicles. J mech. 2010;26(3):317–25. doi: 10.1017/s1727719100003877

[50] Shan W, Song Y. Investigations on formation mechanisms of out-of-round wheel and its influences on the vehicle system. IOP Conf Ser: Mater Sci Eng. 2018;397:012050. doi: 10.1088/1757-899x/397/1/012050

[51] Heinrich G, Klüppel M. Rubber friction, tread deformation and tire traction. Wear. 2008;265(7–8):1052–60. doi: 10.1016/j.wear.2008.02.016

[52] Mukherjee MD. Effect of pavement conditions on rolling resistance. Am. J. Eng. Res. 2014.

[53] Suyabodha A. A relationship between tyre pressure and rolling resistance force under different vehicle speed. MATEC Web Conf. 2017;108:12004. doi: 10.1051/matecconf/201710812004

[54] Tielking JT, Roberts FL. Tire contact pressure and its effect on pavement strain. J Transp Eng. 1987;113(1):56–71. doi: 10.1061/(ASCE)0733-947X(1987)113:1(56

