# Supplementary material for: Tire Deformation-Based Regulation of Braking Torque in Manual Wheelchairs Equipped with Reverse Locking Modules
Source: PLoS One. 2025 Jun 17;20(6):e0325504. doi: 10.1371/journal.pone.0325504 (PMC12173240; doi:10.1371/journal.pone.0325504)
Supplement: S1 Appendix A — Related to Figure 9. (PDF) [file pone.0325504.s001.pdf]

Appendix A – Tables with detailed numerical results of the conducted measurements

Tabl. 1. Matrix of sliding force FZ values depending on the wheelchair inclination angle α and tire pressure p for a seat load mass m = 50 kg

F_Z [N]		p [bar]				
		3	4	5	6	7
α [°]	1	9,08±0,39	10,58±0,70	10,67±0,54	11,17±0,79	11,25±0,55
	2	18,67±0,43	19,00±0,81	19,75±0,44	19,83±0,43	21,00±0,66
	3	29,58±0,39	30,00±0,33	30,42±0,61	30,92±0,39	30,92±0,39
	4	39,17±1,08	40,00±1,00	41,67±0,27	41,92±0,39	42,17±0,86
	5	49,50±0,74	50,00±0,47	50,75±0,98	51,75±0,72	52,42±0,90
	6	61,75±0,55	61,75±0,29	62,67±0,72	63,75±0,86	65,42±1,86
	7	70,08±2,16	70,92±0,39	71,25±0,55	75,42±0,77	76,92±1,67
	8	81,50±1,88	82,58±0,39	82,58±0,39	84,75±0,55	89,00±0,74
	9	92,00±1,56	93,33±0,54	93,08±0,39	95,00±0,94	96,67±0,54
	10	100,67±0,86	103,67±0,79	104,67±0,43	105,17±1,08	107,58±0,61

Tabl. 2. Matrix of sliding force FZ values as a function of the wheelchair inclination angle α and tire pressure p, for a seat load mass m = 70 kg

FZ [N]		p [bar]				
		3	4	5	6	7
α [°]	1	12,67±0,43	12,67±2,09	12,83±0,27	13,42±0,61	14,00±0,88
	2	24,33±0,64	27,08±0,39	27,17±0,79	28,75±0,55	30,58±0,39
	3	38,25±0,55	39,33±0,79	40,33±0,54	42,25±1,27	42,50±0,47
	4	53,17±0,54	53,42±0,39	53,75±0,29	54,00±1,10	54,25±0,86
	5	66,42±1,54	67,75±0,55	68,58±0,39	69,25±0,86	69,83±0,54
	6	80,17±0,54	83,17±0,98	82,42±1,12	83,17±0,98	83,75±0,98
	7	94,00±1,05	95,17±0,54	96,67±0,43	98,75±0,55	99,83±1,18
	8	106,50±0,47	109,92±0,47	109,92±0,39	110,25±0,86	112,75±2,07
	9	121,08±0,61	121,58±0,61	120,75±0,29	123,50±0,47	124,33±1,13
	10	134,58±0,39	136,25±0,55	138,75±0,29	139,83±2,06	139,92±1,74

Tabl. 3. Matrix of sliding force FZ values as a function of the wheelchair inclination angle α and tire pressure p, for a seat load mass m = 90 kg.

F_Z [N]		p [bar]				
		3	4	5	6	7
α [°]	1	12,92±0,61	14,92±0,70	17,42±0,61	17,58±0,39	17,75±0,72
	2	29,08±0,39	30,42±0,39	30,58±0,39	31,75±0,55	31,92±0,39
	3	45,83±0,54	46,92±0,77	47,42±0,70	47,67±0,79	48,00±0,57
	4	64,00±0,57	64,92±0,77	65,42±0,61	65,75±0,29	67,50±0,47
	5	81,33±0,54	81,58±0,61	83,83±0,43	83,92±0,61	87,25±0,55
	6	98,42±0,39	100,33±0,54	101,75±0,55	103,00±0,57	103,67±2,73

	7	114,33±0,54	116,00±0,47	117,50±0,66	118,17±0,54	123,75±1,65
	8	132,33±0,43	136,42±0,43	136,42±0,61	136,83±0,43	139,75±0,98
	9	151,67±0,43	151,92±0,39	154,42±1,30	154,58±0,39	158,50±0,47
	10	167,25±0,86	168,08±0,77	170,92±0,77	174,00±0,57	175,33±1,81
