# Supplementary material for: Tire Deformation-Based Regulation of Braking Torque in Manual Wheelchairs Equipped with Reverse Locking Modules
Source: PLoS One. 2025 Jun 17;20(6):e0325504. doi: 10.1371/journal.pone.0325504 (PMC12173240; doi:10.1371/journal.pone.0325504)
Supplement: S2 Appendix B — Related to Figures 12, 14, 15, and 17. (PDF) [file pone.0325504.s002.pdf]

Appendix B – Graphs of the functions of the developed mathematical models

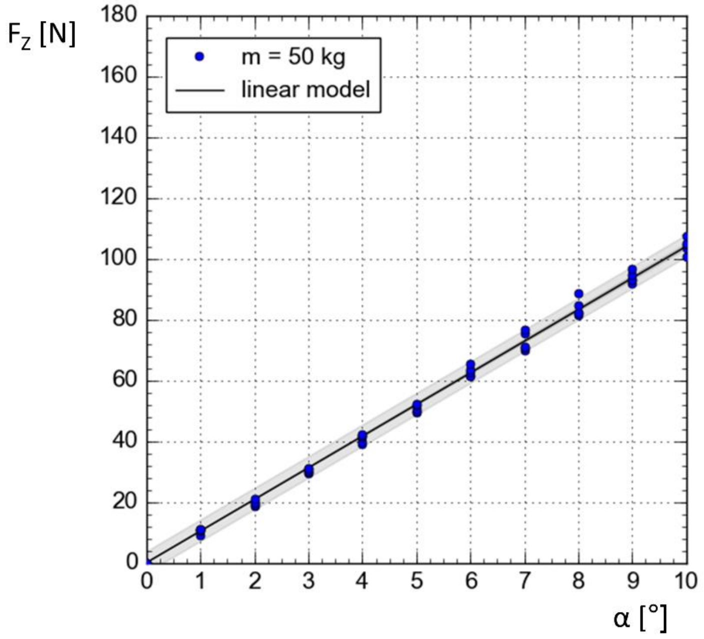

Fig. 5. Graph presenting the experimental results of the sliding force F_Z for tire pressures ranging from 3 to 7 bar, including the analytical model as a function of the slope angle α, for a user mass m = 50 kg. The graph includes the confidence band and prediction band for a confidence level of p = 0.05.

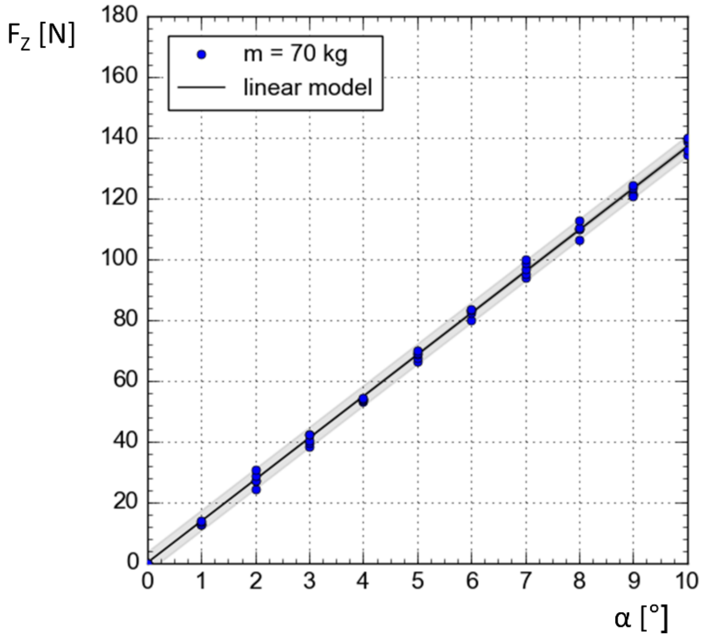

Fig. 6. Graph presenting the experimental results of the sliding force F_Z for tire pressures ranging from 3 to 7 bar, including the analytical model as a function of the slope angle α, for a user mass m = 70 kg. The graph includes the confidence band and prediction band for a confidence level of p = 0.05.

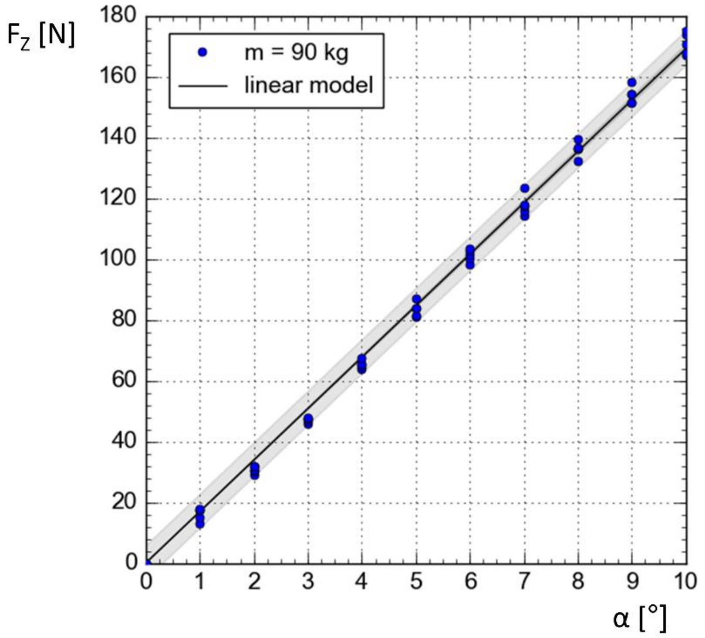

Fig. 7. Graph presenting the experimental results of the sliding force F_Z for tire pressures ranging from 3 to 7 bar, incorporating the analytical model dependent on the slope angle α, for a user mass of m = 90 kg. The graph includes both the confidence band and the prediction band for a confidence interval of p = 0.05.

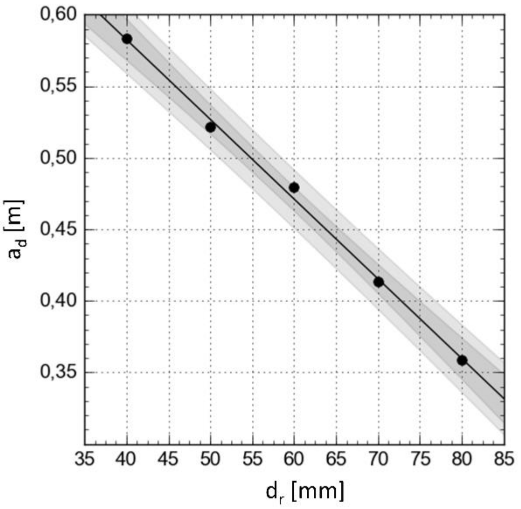

Fig. 12. Actual characteristic of the change in the slope coefficient ad as a function of roller diameter d_r. The graph includes the confidence band and prediction band for a confidence level of p = 0.05 and a sample size of n = 5

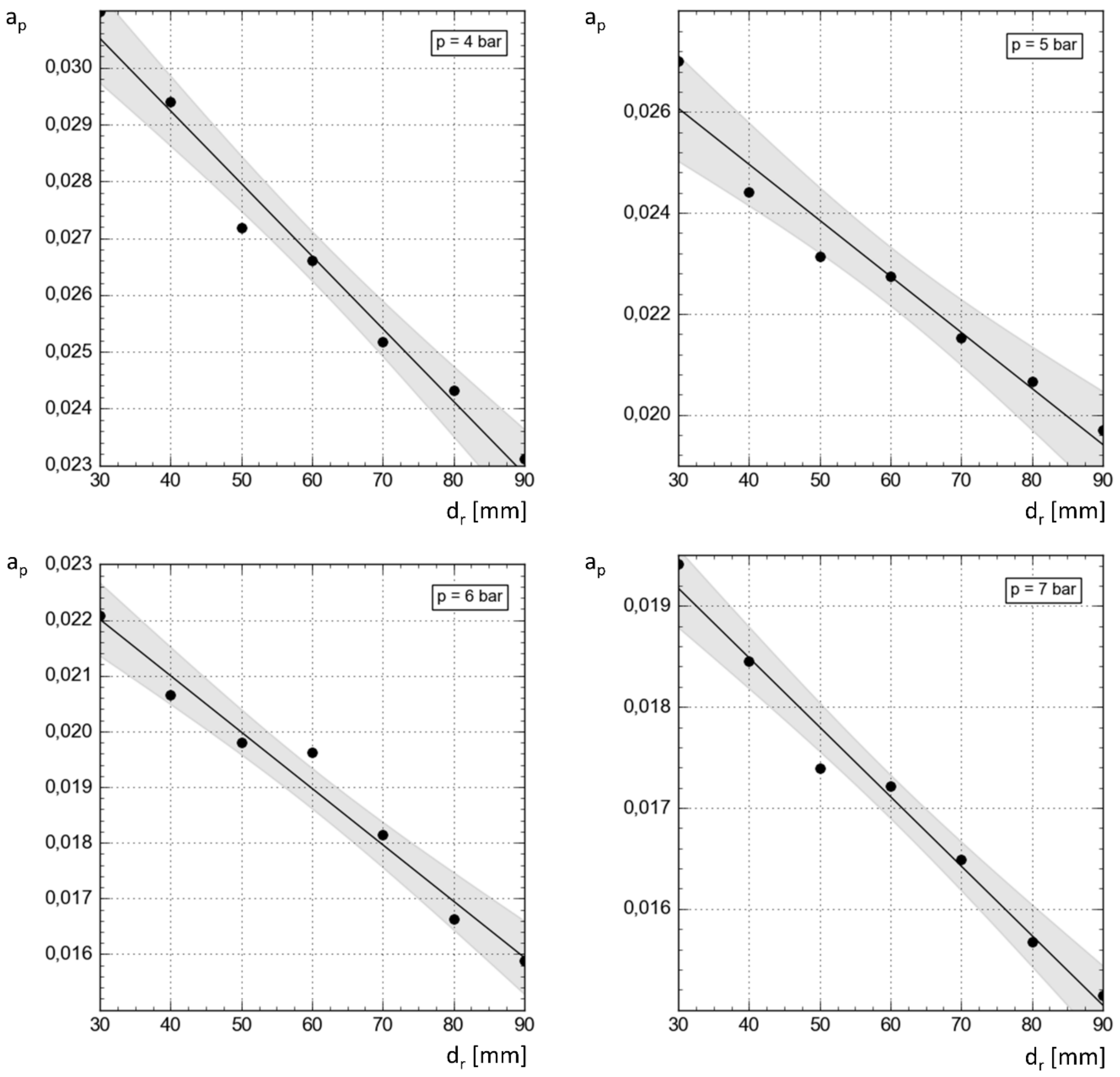

Fig. 15. Trend line graphs with marked confidence bands describing the variation of the slope coefficient ap at the tested roller diameters d_r of the reverse locking module and constant tire pressure p.
